# SARS-CoV-2 Nsp15 endoribonuclease subverts host defenses to enhance viral fitness in lung cells

**DOI:** 10.1128/jvi.01175-25

**Published:** 2025-08-21

**Authors:** Xiang Chi, Xueying Liang, Kishore Vaddadi, Xiaoming Zhang, Chaitanya Gandikota, Sunil More, Lin Liu, Xufang Deng

**Affiliations:** 1 Department of Physiological Sciences, College of Veterinary Medicine, Oklahoma State University 7618, Stillwater, Oklahoma, USA; 2 Oklahoma Center for Respiratory and Infectious Diseases, Oklahoma State University 7618, Stillwater, Oklahoma, USA; 3 Department of Physiological Sciences, The Lundberg-Kienlen Lung Diseases and Infection Laboratory, Oklahoma State University 7618, Stillwater, Oklahoma, USA; 4 Department of Veterinary Pathobiology, College of Veterinary Medicine, Oklahoma State University 7618, Stillwater, Oklahoma, USA; Loyola University Chicago - Health Sciences Campus, Maywood, Illinois, USA

**Keywords:** coronavirus, SARS-CoV-2, nonstructural protein 15, endoribonuclease, immune antagonism

## Abstract

Coronaviruses encode nonstructural protein 15 (Nsp15), a conserved endoribonuclease (EndoU) known to suppress host antiviral responses. It is imperative to understand the role of Nsp15/EndoU during severe acute respiratory syndrome coronavirus 2 (SARS-CoV-2) infection in its primary target respiratory epithelial cells. Using a recombinant wild-type SARS-CoV-2 (rWT) and a mutant with catalytically inactive EndoU (rH234A), we show that the EndoU activity is dispensable for viral replication in Vero cells but critically facilitates immune suppression and infection in physiologically relevant human lung cells, including human lung-derived epithelial cell lines, primary bronchial air-liquid interface cultures, and alveolar type 2 organoids derived from induced pluripotent stem cells. Compared to rWT, rH234A infection elicited augmented type I and III interferon (IFN) responses and stronger RNase L activation, suggesting that Nsp15 suppresses these antiviral pathways during SARS-CoV-2 infection. Although rH234A infection increased PKR phosphorylation, downstream eIF2α phosphorylation and stress granule formation remained limited, implying incomplete PKR/eIF2α pathway activation. Furthermore, Nsp15 mutants lacking key residues required for hexamerization or double-stranded RNA binding also showed impaired replication and heightened IFN responses, highlighting the importance of these structural features for EndoU function. *In vivo*, rH234A was attenuated in K18-hACE2 mice, resulting in improved survival, reduced viral loads, and milder lung pathology. Together, these findings demonstrate SARS-CoV-2 Nsp15/EndoU as a key immune antagonist that facilitates lung infection, underscoring its potential as a target for antiviral development.

**IMPORTANCE:**

Severe acute respiratory syndrome coronavirus 2 (SARS-CoV-2) infects upper and lower airway epithelial cells, and the latter infection can result in severe pneumonia. Understanding how the virus evades host immunity in these primary target cells is critical for developing effective therapies. Nsp15, a conserved coronavirus endoribonuclease (EndoU), is known to suppress antiviral responses, but its precise role in respiratory epithelial cells has remained unclear. This study reveals that while EndoU activity is dispensable for viral replication, it is essential for dampening host defenses in human lung-derived epithelial cell lines, primary bronchial air-liquid interface cultures, and alveolar type 2 organoids. Loss of EndoU activity results in enhanced interferon signaling and RNase L activation, along with partial PKR pathway engagement. Structural integrity, including double-stranded RNA binding and hexamerization, is vital for Nsp15 function. *In vivo*, an EndoU-deficient virus is attenuated in K18-hACE2 mice. These findings position Nsp15 as a key immune antagonist and a promising target for antiviral intervention.

## INTRODUCTION

Coronavirus (CoV) virions carry positive-sense, single-stranded RNA genomes, ranging from approximately 26 to 30 kb in length. The genomic RNA serves as messenger RNA (mRNA) and is translated into two large polyproteins, pp1a and pp1ab. The polyprotein pp1ab is an extended form of pp1a, produced through a −1 ribosomal frameshift mechanism. This polyprotein is subsequently cleaved by two viral proteases into 16 nonstructural proteins (Nsps), which play essential roles in viral RNA synthesis and reshaping a favorable cellular environment for viral replication (1). Among these 16 Nsps, Nsp15 is the 15th cleavage product of pp1ab, counting from the amino terminus to the carboxyl terminus of pp1ab (2). It possesses an endoribonuclease (EndoU) domain with a distinct preference for cleaving pyrimidine-containing RNA sequences (U/C) (3, 4). Biochemical and structural studies have documented that Nsp15 monomers form hexamers, which is the fully active form of EndoU that binds to RNA substrates and executes optimal EndoU activity (5, 6). Nsp15 was initially assumed to be directly involved in viral RNA synthesis due to its colocalization with newly synthesized viral RNA and other viral proteins that are known to be involved in viral RNA synthesis, such as Nsp8 and Nsp12 (7–9). However, subsequent studies using recombinant mutant viruses expressing a catalytic-inactive Nsp15 revealed that the EndoU activity is dispensable for viral RNA synthesis but is critical for evading host double-stranded (ds) RNA-induced antiviral responses (10–12).

Many viruses, including CoVs, produce dsRNA intermediates or byproducts during replication (13). These viral dsRNAs are recognized by host cells as non-self RNA species (14). Eukaryotic cells express several pattern recognition receptors (PRRs) to recognize viral dsRNA and initiate potent antiviral responses. For instance, RIG-I-like PRRs such as RIG-I and MDA5 detect dsRNA molecules and activate the transcription of type I interferons (IFNs) (e.g., IFN-β) and type III IFNs (e.g., IFN-λ1). These IFNs are secreted from cells and bind to specific cell surface receptors, initiating the formation and activation of the interferon-stimulated gene factor 3 (ISGF3) complex that is composed of the signal transducer and activator of transcription 1 (STAT1), STAT2, and IFN regulatory factor 9 (IRF-9). This complex, in turn, mediates the transcriptional activation of numerous ISGs. Among these ISGs, several act as dsRNA sensors, such as the 2´-5´-oligoadenylate synthetases (OAS1−3) and protein kinase R (PKR), which play crucial roles in initiating immediate antiviral responses. In human cells, OAS3 specifically binds to dsRNA, undergoing a conformational change that activates its enzymatic activity to synthesize 2´-5´-oligoadenylates (2-5A) (15). The 2-5A molecules subsequently bind to and activate the host endoribonuclease RNase L, which executes a robust antiviral response by catalyzing global RNA degradation, including ribosomal RNAs (rRNAs) required for viral protein synthesis. Extensive RNA degradation releases host RNA-binding proteins (RBPs), such as poly-A binding protein cytoplasmic 1 (PABPC1), promoting PABPC1 to associate with cytoplasmic granules and translocate to the nucleus (16–18). PABPC1 nuclear translocation is RNase L-dependent, serving as a hallmark of RNase L activation (16, 18). In the nucleus, these RBPs impede the nuclear export of host mRNAs, effectively limiting antiviral proteins translation and further hindering viral gene expression (16, 19). PKR, another cytoplasmic dsRNA sensor, binds to dsRNA, triggering its autophosphorylation and subsequent activation. Activated PKR phosphorylates eukaryotic initiation factor 2α (eIF2α), thereby inducing the integrated stress response and halting global protein translation, which represents a fast defense mechanism to curtail viral replication (20, 21).

We and others have previously reported that the Nsp15/EndoU activity prevents the activation of dsRNA-induced antiviral pathways. Recombinant murine coronaviruses, mouse hepatitis virus (MHV), expressing a catalytically inactive Nsp15 (H262A or H277A) induced early and robust activation of the MDA5-mediated type I IFN, OAS/RNase L, and PKR/eIF2α signaling pathways in mouse bone marrow-derived macrophages (BMDMs) and exhibited a markedly replication defect in both BMDMs and mice (11, 12). The replication defect of the Nsp15 mutant virus was rescued when the Nsp15 mutant virus replicated in IFN α/β receptor-knockout BMDMs and mice, suggesting its critical role in antagonizing the host antiviral immunity. A similar antagonistic phenotype has been reported for Nsp15 mutants of human CoV 229E strain (HCoV-229E, α-CoV), porcine epidemic diarrhea virus (PEDV, α-CoV), Middle East respiratory syndrome CoV (MERS-CoV, β-CoV), infectious bronchitis virus (IBV, γ-CoV), and porcine deltacoronavirus (PDCoV, δ-CoV), implying that Nsp15/EndoU is a highly conserved immune antagonist across CoVs (12, 22–26). Later studies revealed that Nsp15 could cleave viral genomic RNA or degrade polyU from the 5´ end of negative-stranded RNA to limit the accumulation of viral dsRNA molecules (27, 28).

In light of the well-characterized functions of Nsp15/EndoU in other CoVs, severe acute respiratory syndrome coronavirus 2 (SARS-CoV-2) Nsp15 has been a study focus since the coronavirus disease 2019 (COVID-19) pandemic began. Many *in vitro* biochemical and structural characterizations have revealed the cleavage preference and RNA-binding capacity of SARS-CoV-2 Nsp15 (6, 29–33). Although the results obtained from ectopic overexpression experiments suggested that SARS-CoV-2 Nsp15 was an immune antagonist (34, 35), fewer studies investigated the role of Nsp15 in SARS-CoV-2 infection in primary human lung cells and *in vivo* animal models. In the present study, we generated recombinant SARS-CoV-2 mutant viruses and found that the EndoU activity was critical for SARS-CoV-2 infection in the human lung cells, including human lung-derived cell lines, primary bronchial epithelial air-liquid interface (ALI) cultures, and induced pluripotent stem cell (iPSC)-derived alveolar type 2 epithelial cell (AT2) organoids. Our results indicate that Nsp15/EndoU antagonizes type I and III IFN response and the OAS/RNase L pathway. Moreover, we demonstrate that the Nsp15 mutant virus is attenuated in transgenic mice expressing human angiotensin-converting enzyme (K18-hACE2), exhibiting reduced lung viral burden, milder lung pathology, and improved survival. These findings underscore the critical role of Nsp15/EndoU in SARS-CoV-2 pathogenesis *in vivo*.

## RESULTS

### Nsp15 EndoU activity is dispensable for SARS-CoV-2 replication in Vero cells

**Fig 1:**
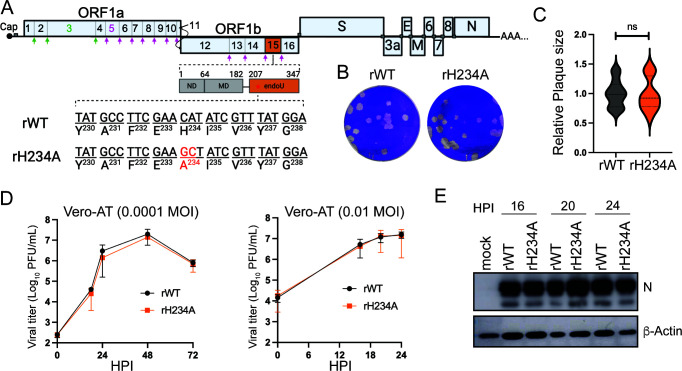
The EndoU activity of SARS-CoV-2 Nsp15 is dispensable for viral replication. (**A**) Schematic diagram of the SARS-CoV-2 genome. The open reading fram 1a (ORF1a) and ORF1b are cleaved by two viral proteases Nsp3 (green) and Nsp5 (purple). Full-length Nsp15 is shown with an N-terminal domain (ND), a middle domain (MD), and the EndoU domain. Nucleotide and amino acid changes are shown in red. (**B**) Representative plaque images in Vero-AT cells at 48 hours post-infection (HPI). (**C**) Plaque size was compared using unpaired *t*-test with Welch’s correction (*n* = 15, *P* = 0.8714). ns, not significant. (**D**) Viral growth curves in Vero-AT cells with two inoculation doses (0.0001 or 0.01 multiplicity of infection [MOI]). Data in **D** are shown as mean ± SD (*n* = 3). (**E**) Western blotting of viral N protein in Vero-AT cells that were infected with 0.01 MOI of rWT or rH234A. β-Actin served as a loading control. Data in this figure are representative of at least two independent experiments.

The EndoU domain of SARS-CoV-2 spans the 207−347 amino acid residues of Nsp15, containing two conserved catalytic histidine residues (H) at 234 and 249. Alanine substitution of these histidine residues abolished 99% of the ribonucleolytic activity of Nsp15 (6). To determine the role of Nsp15 EndoU activity in viral replication and pathogenesis, we generated a recombinant SARS-CoV-2 encoding a catalytic-inactive Nsp15 by substituting H234 with alanine (denoted rH234A) using an infectious clone of the SARS-CoV-2 Washington strain 1 (WA1) (36). An isogenic recombinant WA1 was also rescued to serve as wild-type (WT) control (denoted rWT) (Fig. 1A).

We first compared the replication of two viruses by measuring their plaque sizes and growth kinetics in a Vero-E6 cell line expressing hACE2 and human transmembrane protease serine 2 (hTMPRSS2) (Vero-AT), a widely used IFN-deficient cell type for SARS-CoV-2 propagation (37, 38). As shown in Fig. 1B and C, both viruses formed clear-edge, round plaques with similar sizes at 48 hours post-infection (HPI). Growth kinetic assays revealed that both viruses exhibited comparable replication kinetics in Vero-AT cells at two different multiplicities of infection (MOIs), 0.0001 and 0.01 (Fig. 1D), indicating a dispensable role of the EndoU activity in SARS-CoV-2 replication. In line with this result, our western blot results show no marked differences in the nucleocapsid (N) expression between two viruses in Vero-AT cells (Fig. 1E). Collectively, these results demonstrate that the genetic ablation of Nsp15 EndoU activity does not impair SARS-CoV-2 replication in the IFN-deficient Vero cells.

### Nsp15 EndoU activity promotes SARS-CoV-2 replication in human lung-derived cell lines

**Fig 2:**
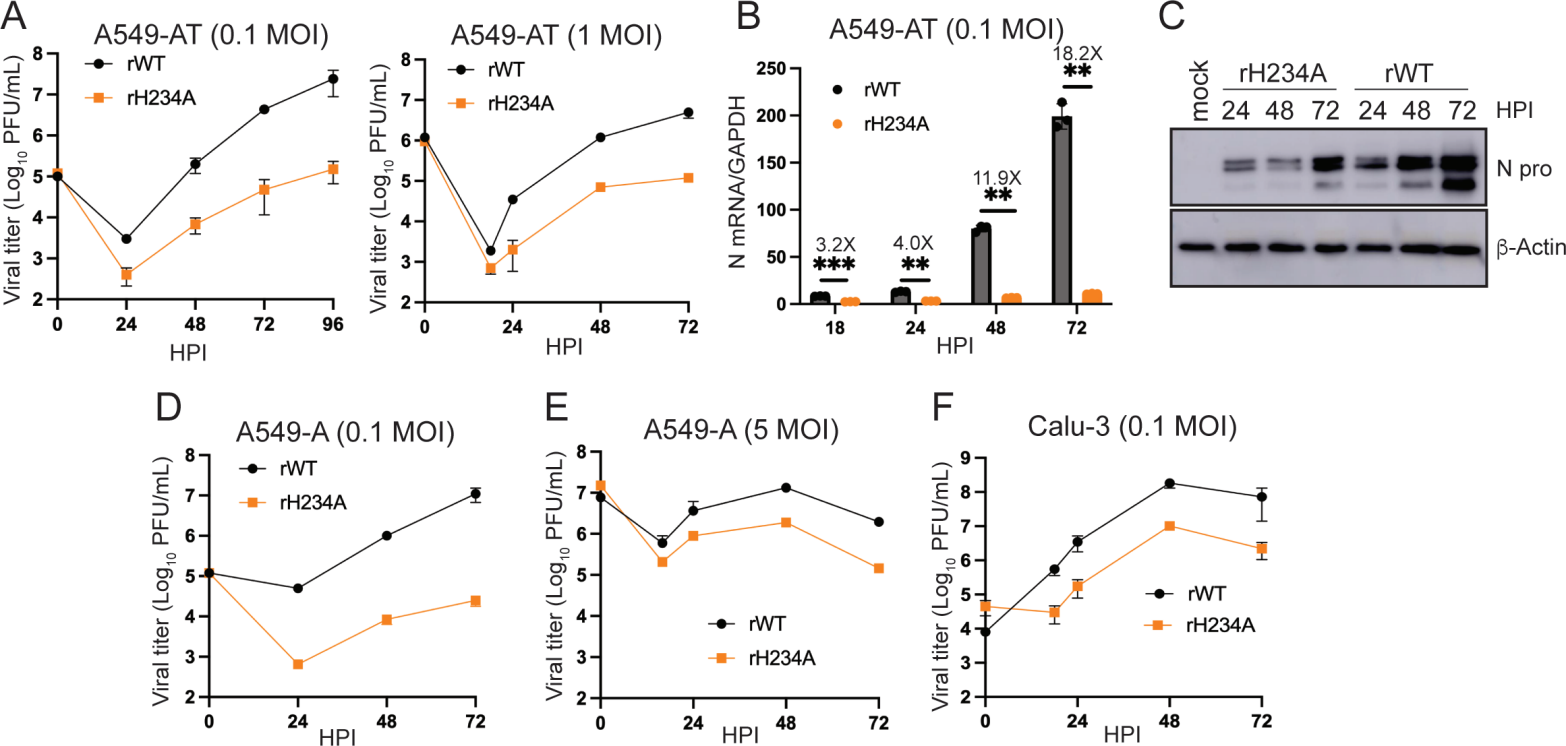
rH234A exhibited impaired replication in human lung-derived epithelial cells. (**A and B**) A549-AT cells were infected with rWT or rH234A at 0.1 and 1 MOI. The culture supernatants were collected for extracellular viral titration (**A**). Whole cell lysates were collected for RNA extraction and subsequent quantification of viral N mRNA level (**B**). Data in A and B were shown as mean ± SD (*n* = 3), and data in **B** were analyzed with a two-way analysis of variance Tukey’s multiple comparison test. **, *P* < 0.01; ***, *P* < 0.001. (**C**) Western blotting of viral N protein in A549-AT cells (0.1 MOI). β-Actin was served as a loading control. (**D−F**) Growth curves of rWT and rH234A in A549-A (0.1 or 5 MOI) or Calu-3 cells (0.1 MOI). Data in this figure are representative of at least two independent experiments. Data in **D−F** are shown as mean ± SD (*n* = 3).

We next assessed the impact of the EndoU activity on SARS-CoV-2 replication in more physiologically relevant cell types. To determine viral growth kinetics, we first used a human lung epithelium-derived A549 cell line that was lentiviral-transduced to stably express hACE2 and hTMPRSS2 (denoted A549-AT) (39). We found that rH234A exhibited a significant replication defect compared to rWT in this cell line. First, growth kinetics assays revealed rH234A had 1−2 log lower extracellular viral titers during the exponential growth phase compared to the isogenic rWT when two different inoculation doses (0.1 and 1 MOI) were used (Fig. 2A). Second, RT-qPCR analysis of the intracellular N gene results showed rH234A had significantly lower N mRNA levels than rWT at all tested time points (Fig. 2B). Third, the western blot results indicated rH234A had relatively lower N protein level compared to rWT (Fig. 2C). We also compared the replication of rWT and rH234A in a different A549 cell line that stably expressed hACE2 (denoted A549-A) through transposon-mediated gene insertion. Again, a marked replication defect was observed for rH234A compared to rWT when an inoculation dose of 0.1 MOI was used (Fig. 2D). This viral titer difference between the two viruses was less pronounced (3 ~ 10-fold) when a high inoculation dose of 5 MOI was used (Fig. 2E). We next used Calu-3 cells, a human lung-derived epithelial cell line that endogenously expresses ACE2 and TMPRSS2 and is naturally permissive to SARS-CoV-2 infection. It has been widely used for assessing SARS-CoV-2 replication and host antiviral responses (40, 41). Using this cell line, we again observed that rH234A exhibited extracellular viral titers approximately 1 log lower than rWT at the indicated time points (Fig. 2F). Collectively, these results demonstrate that mutating the catalytic H234 residue impairs viral replication, suggesting that the EndoU activity promotes SARS-CoV-2 infection in these human lung-derived epithelial cell lines.

### Nsp15 EndoU activity is critical for optimal SARS-CoV-2 replication in primary human lung epithelial cells

**Fig 3:**
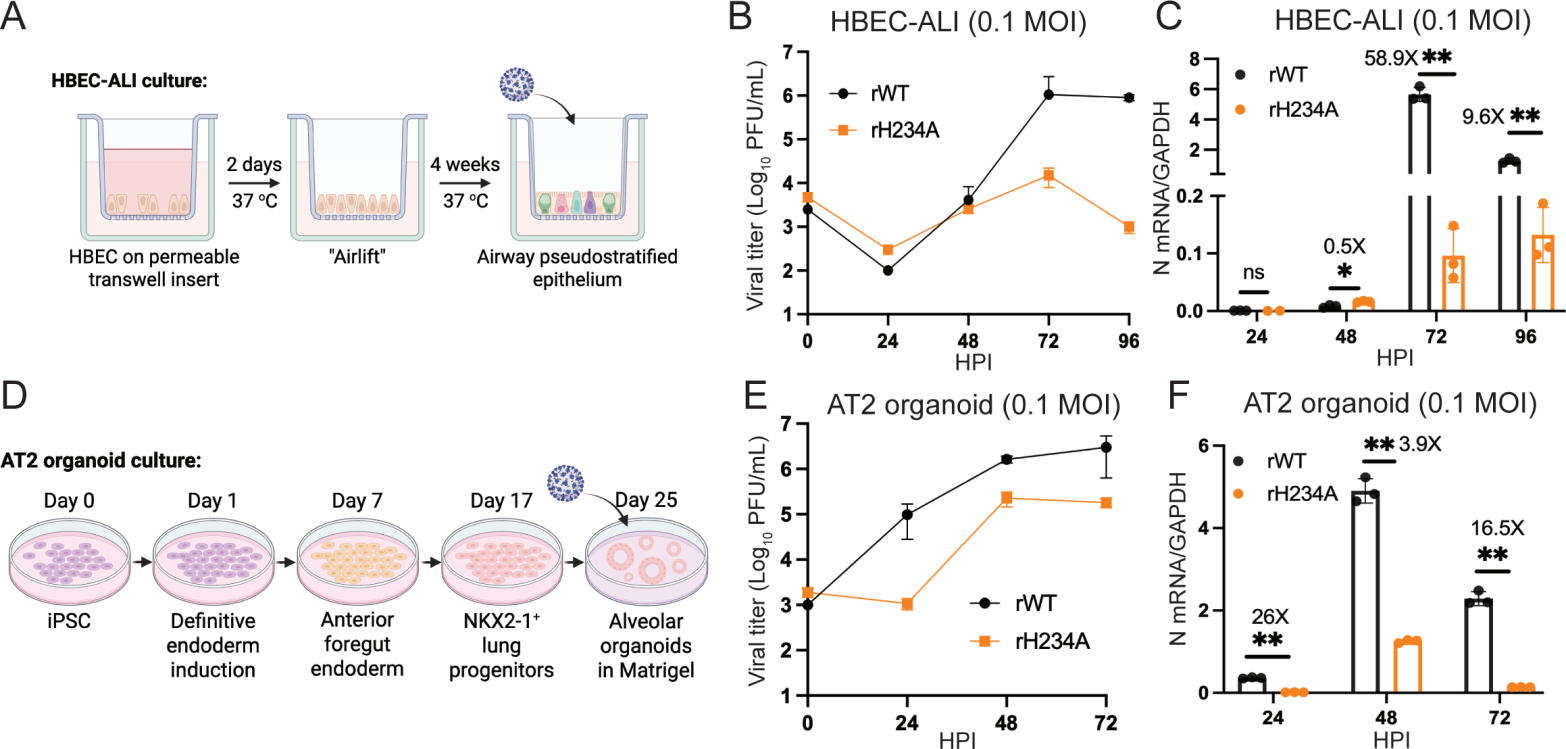
rH234A exhibited impaired replication in primary human lung epithelial cells. (**A**) Schematic diagram of HBEC differentiation. (**B and C**) Differentiated HBECs in Transwell inserts were inoculated with rWT or rH234A at 0.1 MOI. (**B**) The apical mucus layers were collected for viral titration, and (**C**) the cells on the membrane were harvested for viral N mRNA quantification. (**D**) Schematic diagram of alveolar type 2 organoids induction and differentiation. (**E and F**) AT2 organoids grown in Matrigel were inoculated with rWT or rH234A at 0.1 MOI. (**E**) The culture Matrigel content was collected for viral titration. (**F**) The organoids were harvested for viral N mRNA quantification. Data in this figure are representative of two independent experiments and shown as mean ± SD (*n* = 3). Data in **C and F** were analyzed with a two-way analysis of variance Tukey’s multiple comparison test. *, *P* < 0.05; **, *P* < 0.01. The images in **A** and **D** were prepared using Biorender.com.

Next, we extended our evaluation with two primary human lung epithelial cultures, normal human bronchial epithelial cells (HBECs) and alveolar type II epithelial cells (AT2). The HBECs were differentiated on Transwell inserts to establish ALI cultures for 4 weeks and then subjected to viral infections (Fig. 3A). Growth kinetic analysis revealed that rWT propagated efficiently in the HBEC-ALI cultures, reaching titers of approximately 10^6^ plaque-forming units (PFU) per milliliter by 72 HPI. rH234A exhibited similar extracellular titers to rWT at 24 and 48 HPI, but showed more than a 2-log reduction in viral titers at 72 and 96 HPI (Fig. 3B). The extracellular titer results were corroborated by the RT-qPCR results of the viral N gene, showing that rH234A had a significantly lower N gene level than rWT at 72 and 96 HPI (Fig. 3C). Next, AT2 organoids were derived from iPSCs and grown in Matrigel as we previously reported (42) (Fig. 3D). After differentiation, the organoids were harvested from Matrigel and used for viral infection. Consistent with the results described above, we observed markedly lower viral titers (1 ~ 2 logs) (Fig. 3E) and N gene levels (Fig. 3F) in the rH234A-infected AT2 organoids compared to the rWT-infected controls. Collectively, these results demonstrate a marked replication impairment of rH234A compared to rWT, suggesting a critical role of Nsp15 EndoU activity in SARS-CoV-2 infection in primary human lung epithelial cells.

### SARS-CoV-2 Nsp15 antagonizes type I and III IFN activation

**Fig 4:**
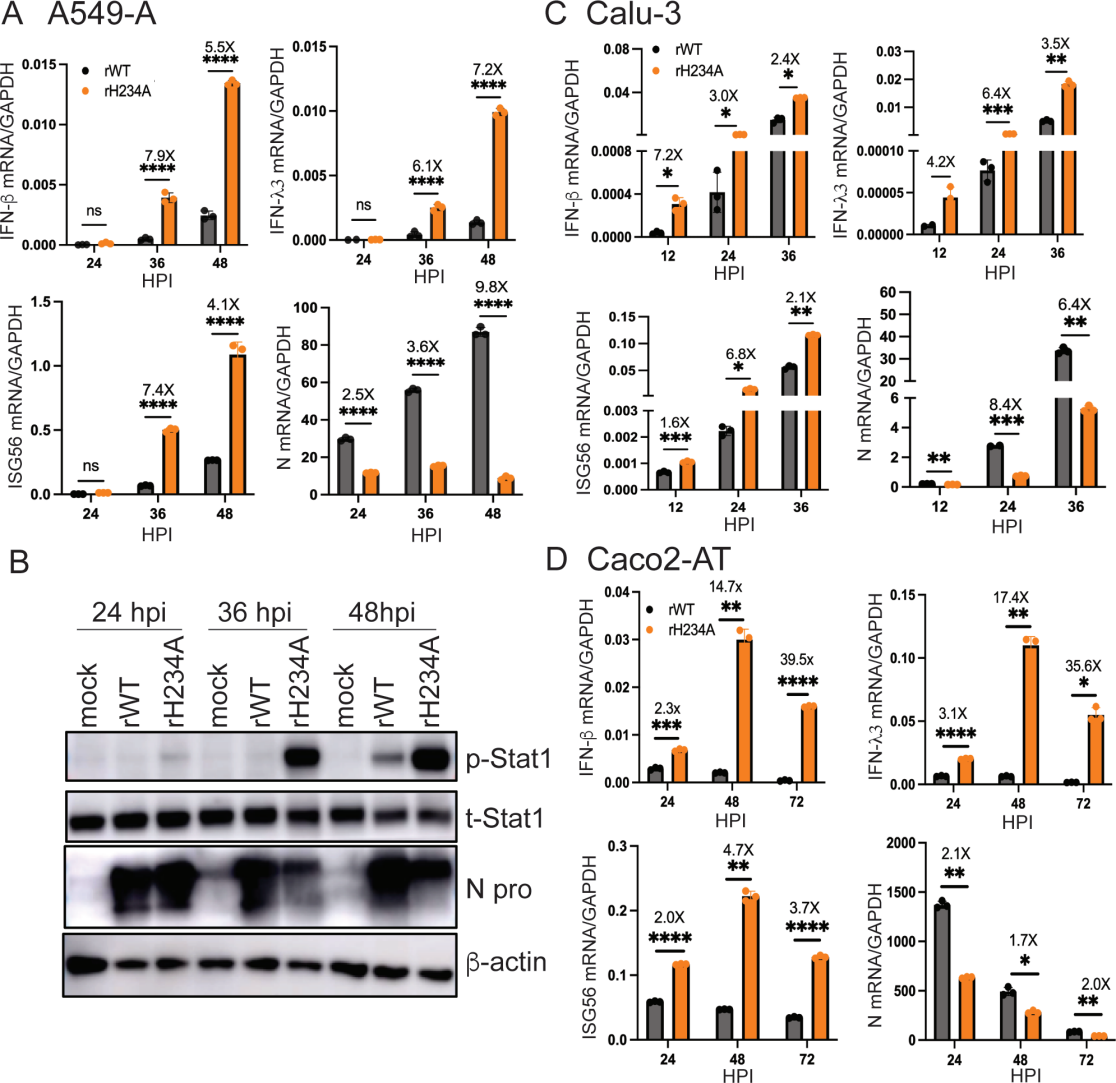
SARS-CoV-2 Nsp15 antagonizes type I and III IFN activation. (**A**) Quantification of *IFN-*β, *IFN-*λ*3*, *ISG56* mRNA, and viral N gene in A549-A cells infected with 5 MOI of rWT or rH234A by RT-qPCR. (**B**) Immunoblotting phosphorylated Stat1 (p-Stat1), total Stat1 (t-Stat1), viral N protein (N pro), and β-actin of A549-A cells infected with 5 MOI of rWT or rH234A. (**C**) RT-qPCR quantification of *IFN-*β, *IFN-*λ*3*, *ISG56* mRNA, and viral N gene in Calu-3 cells infected with 1 MOI of rWT or rH234A. (**D**) RT-qPCR quantification of *IFN-*β, *IFN-*λ*3*, *ISG56* mRNA, and viral N gene in Caco2-AT cells infected with 0.1 MOI of rWT or rH234A. Data in **A**, **C**, and **D** are shown as mean ± SD (*n* = 3) and were analyzed with a two-way analysis of variance Tukey’s multiple comparison test. Data in this figure are representative of at least two independent experiments. *, *P* < 0.05; **, *P* < 0.01; ***, *P* < 0.001; ****, *P* < 0.0001.

We and others have previously reported that the EndoU activity of Nsp15 in several CoVs suppresses type I and III IFN responses (11, 12, 23, 25, 26). To determine whether SARS-CoV-2 Nsp15 similarly antagonizes type I and III IFN activation, we measured the mRNA levels of *IFN-*β (type I), *IFN-*λ*3* (type III), and *ISG56* in A549-A cells following infection. Viral N gene expression was measured as an indicator of intracellular viral replication. At 36 and 48 HPI, rH234A-infected cells showed significantly elevated mRNA levels of *IFN-*β (7.9- and 5.5-fold), *IFN-*λ*3* (6.1- and 7.2-fold), and *ISG56* (7.4- and 4.1-fold), respectively, compared to rWT infection. This increase occurred despite a 3.6- and 9.8-fold reduction in N gene expression at the same time points (Fig. 4A). These results suggest that rH234A infection triggers a more robust IFN response in A549-A cells. We further performed immunoblotting assays to determine the phosphorylation of Stat1 (p-Stat1), a hallmark of type I/III IFN signaling activation. As shown in Fig. 4B, p-Stat1 was readily detected in rH234A-infected cells starting at 24 HPI, with pronounced levels at 36 and 48 HPI. In contrast, rWT infection induced weaker and delayed p-Stat1 detection. The N protein level of rH234A infection was noticeably lower than that of rWT infection at 36 and 48 HPI, aligning with the N gene mRNA levels determined by RT-qPCR (Fig. 4A).

To further support this conclusion, we performed similar qPCR assays in Calu-3, HBECs, and Caco2-AT cells, a human colorectal epithelial cell line stably expressing hACE2 and hTMPRSS2 (43). In Calu-3 cells, rH234A infection induced significantly higher levels of *IFN-*β*, IFN-*λ*3,* and *ISG56* at 12, 24, and 36 HPI, despite markedly reduced N gene expression (Fig. 4C). In HBEC-ALI cultures infected at an MOI of 0.1, rH234A led to a modest (fourfold) increase in *IFN-*β and *IFN-*λ*3* at 72 HPI and levels only slightly lower than those induced by rWT at 96 HPI, despite distinctly lower N gene levels (31-fold and 65-fold, respectively) at these time points (Fig. S1). In Caco2-AT cells, rH234A infection consistently resulted in robust upregulation of all three IFN-related genes at all tested time points (Fig. 4D). Collectively, these results demonstrate that rH234A infection triggers a stronger type I/III IFN response, supporting the conclusion that SARS-CoV-2 Nsp15 EndoU activity suppresses the host IFN pathways.

### The H14 and W332 residues are important for Nsp15-mediated IFN antagonism

**Fig 5:**
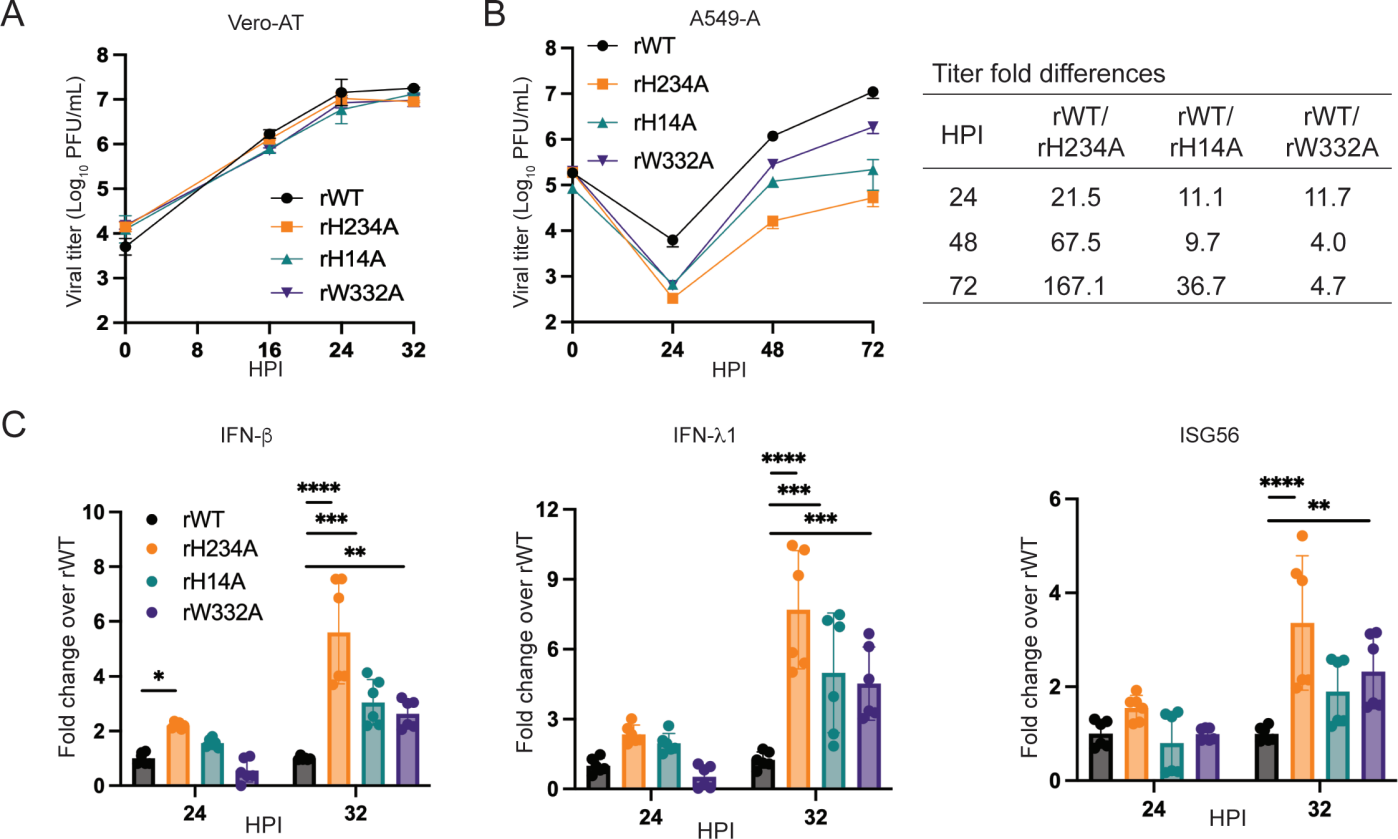
The H14 and W332 residues are important for Nsp15-mediated IFN antagonism. (**A**) Growth kinetic curves of recombinant viruses in Vero-AT cells (0.01 MOI). (**B**) Growth curves (left) and titer fold difference (right) of recombinant viruses in A549-A cells (0.1 MOI) (right). (**C**) RT-qPCR quantification of *IFN-*β, *IFN-*λ*1*, and *ISG56* mRNA in Caco2-AT cells infected with 0.1 MOI of indicated recombinant virus. Data in **A** and **B** are representative of at least two independent experiments and are shown as mean ± SD (*n* = 3-6). Data in **C** are the pooled results of two independent experiments, shown as mean ± SD (*n* = 4-6), and were analyzed with a two-way analysis of variance Tukey’s multiple comparison test. *, *P* < 0.05; **, *P* < 0.01; ***, *P* < 0.001; ****, *P* < 0.0001.

Next, we examined the impact of Nsp15 hexamerization and dsRNA binding besides its catalytic activity on viral replication and Nsp15 EndoU-mediated immune suppression. Similar to other CoVs, SARS-CoV-2 Nsp15 forms hexamers *in vitro* to execute the EndoU activity (6). Mutating key residues (e.g., histidine-14, H14) within the N-terminal domain of Nsp15 greatly disrupted its hexamerization and consequently abolished its EndoU activity (29). A recent report argues that the hexamerization is an artifact due to the N-terminal epitope tags that are typically fused with Nsp15 to facilitate protein expression and purification in bacteria (44). Whether Nsp15 hexamerization is required for EndoU activity in the context of viral infection remains unclear. On the other hand, several studies have indicated that Nsp15/EndoU prevents viral dsRNA accumulation likely through physical binding and cleavage of dsRNA (27, 28). A cryo-electron microscopy study revealed that the tryptophan-332 (W332) residue is critical for dsRNA binding, as alanine substitution at this position reduced dsRNA cleavage by 75% (31). Thus, it is imperative to determine the impact of Nsp15 dsRNA binding during viral infection. To this end, we generated two additional recombinant Nsp15 mutant viruses, one harbors an H14A mutation (denoted rH14A) and the other one carries a W332A mutation (denoted rW332A). In Vero-AT cells, similar to rH234A, both rH14A and rW332A exhibited comparable growth kinetics as rWT with less than threefold titer differences at the tested time points (Fig. 5A; Fig. S2A). We also assessed viral replication in Vero-E6 cells and found all four viruses have similar growth kinetics (<5-fold titer differences) (Fig. S2B). These results suggest that these mutations do not significantly impair viral replication in Vero cells. In A549-A cells, however, rH14A and rW332A exhibited noticeably impaired growth kinetics compared to rWT (Fig. 5B). The growth impairment of rH14A and rW332A is intermediate compared to rH234A. We also investigated the immune antagonistic effects of rH14A and rW332A in Caco2-AT cells and found these mutants exhibited an intermediate phenotype between rWT and rH234A. As shown in Fig. 5C, infection of rH14A and rW332A stimulated significantly higher levels of type I/III IFNs and ISG56 mRNA than rWT infection but modestly lower than rH234A infection. The intermediate phenotype in replication and immune suppression of rH14A and rW332A coincides with the relative EndoU activity of cognate Nsp15 mutants to process RNA substrates (29, 31). While our work is in progress, Evdokimova et al. reported that a different hexamerization-defective SARS-CoV-2 Nsp15 mutant virus (E3A) was attenuated in human lung cells and induced higher expression of ISGs (45). These data together suggest that these residues are important for Nsp15-mediated IFN antagonism, likely through involvement in Nsp15 hexamerization or dsRNA binding. 

### rH234A infection induced enhanced activation of the OAS/RNase L pathway in infected human lung cells

**Fig 6:**
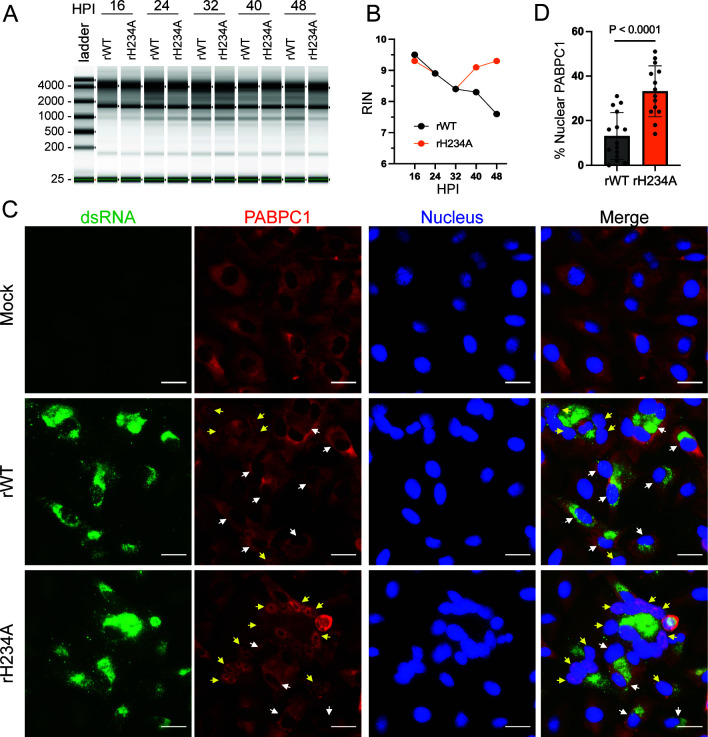
rH234A infection differentially promotes the nuclear trafficking of PABPC1. (**A**) RNA integrity analysis of A549-A cells infected with rWT or rH234A at 5 MOI. The cells were harvested at indicated times for whole RNA extraction and subsequent TapeStation analysis. (**B**) The RIN of the total RNA collected in **A**. (**C**) Immunofluorescence assay (IFA) of viral dsRNA and host PABPC1 protein. A549-A cells were infected with rWT or rH234A at 5 MOI and fixed at 24 HPI. Viral dsRNA and host PABPC1 protein were probed with specific antibodies, and the nucleus was stained with Hoechst 33342. Infected cells with or without PABPC1 nuclear translocation are indicated with yellow- and white-color arrows, respectively. Scale bar: 20 µM. (**D**) Quantification of PABPC1 nuclear localization in virus-infected cells from 14 to 15 views of IFA slides. Data are shown as mean ± SEM (*n* = 14-15) and analyzed using unpaired *t*-test with Welch’s correction. Data in the figure are representative of at least two independent experiments.

 We and others previously reported that the Nsp15/EndoU of several CoVs could antagonize the dsRNA-induced OAS/RNase L and PKR pathways (11, 12, 23–25). To determine whether SARS-CoV-2 EndoU antagonizes OAS/RNase L activation in human lung epithelial cells, we first performed RNA TapeStation analysis of total RNA extracted from A549-A cells following virus infection (MOI 5) to detect rRNA degradation, a hallmark of OAS/RNase L-mediated RNA decay (Fig. 6A). We found that the rRNA integrity number (RIN) of rWT-infected cells gradually declined during the observed infection course, while the rRNA integrity of rH234A-infected cells was similar to that of rWT-infected cells during 16−32 HPI and then rebounded afterward (40−48 HPI) (Fig. 6B). The RIN rebound is likely due to a higher proportion of uninfected cells left for the assay resulting from the significantly impaired replication of rH234A (Fig. 2). We also examined the rRNA integrity in Calu-3 cells and HBECs. Again, we found that the RNA integrity of Calu-3 (Fig. S3A) or HBECs (Fig. S3B) gradually deteriorated upon infection by either virus, although the rRNA decay rate of the rH234A-infected cells was slightly slower than that of rWT-infected cells, which correlates with the replication defect of rH234A in both cell cultures (Fig. 2F and 3B, respectively). These results suggest that, albeit markedly impaired replication, rH234A activates the OAS/RNase L pathway to a similar extent as the rWT infection does during the early phase of SARS-CoV-2 infection.

Since the TapeStation assay analyzes the total RNA from the entire cell population, the rRNA integrity of infected cells may be masked by the intact rRNA from uninfected cells. We indeed found that despite a high MOI of 5 being used, roughly only 54% and 41% of A549-A cells (*P* = 0.0101) were infected by rWT or rH234A, respectively (Fig. S4A and B). To closely assess RNase L activation specifically in infected cells, we performed an immunofluorescence assay (IFA) to examine the nuclear trafficking of PABPC1, a hallmark of RNase L activation (16, 18). To this end, we first determined whether PABPC1 functions properly in A549-A cells upon RNase L activation through examining the localization of PABPC1 following transfection of high-molecular-weight poly(I:C), a viral dsRNA mimic that is known to activate multiple host dsRNA-induced pathways including the OAS/RNase L pathway. As shown in Fig. S4C, we observed robust nuclear accumulation of PABPC1, as indicated by its co-staining with a nuclear dye. In the cytoplasm, PABPC1 formed cytoplasmic puncta that were closely associated with G3BP1, consistent with previous reports (16–18). We next examined the PABPC1 localization during SARS-CoV-2 infection. As shown in Fig. 6C, PABPC1 was mostly detected in the cytoplasm with a diffused pattern in uninfected cells. In contrast, some infected cells showed PABPC1 cytoplasmic puncta and/or nuclear accumulation. To compare RNase L activation, we counted infected cells (dsRNA-positive) from 15 distinct IFA views for each viral infection and calculated the percentage of infected cells exhibiting PABPC1 cytoplasmic puncta or nuclear translocation (Fig. S4A). Among these dsRNA-positive cells, 34% (209/604) of the rH234-infected cells exhibited PABPC1 nuclear accumulation, which is roughly threefold higher than the 13% observed in rWT-infected cells (128/1,005, *P* < 0.0001), suggesting rH234A infection triggering a stronger RNase L activation (Fig. 6D). We also noticed that an average of 44% rWT- and 51% rH234A-infected cells show PABPC1 cytoplasmic puncta, although the percentages do not statistically differ between the two infections (Fig. S5), consistent with the notion that PABPC1 cytoplasmic puncta formation can be RNase L-independent (16, 18).

To determine whether virus-induced PABPC1 nuclear trafficking is RNase L-dependent, we generated a RNase L knockout (RL-KO) A549-A cell line using CRISPR-Cas9 (Fig. S6A). In Cas9 control cells, poly(I:C) treatment induced both cytoplasmic puncta formation and nuclear accumulation of PABPC1, consistent with RNase L activation. In contrast, RL-KO cells exhibited PABPC1 cytoplasmic puncta but no nuclear accumulation upon poly(I:C) stimulation (Fig. S6B), supporting previous findings that RNase L is required for PABPC1 nuclear trafficking (16, 18). Notably, infection with either rWT or rH234A did not induce PABPC1 nuclear trafficking (Fig. S6C), indicating that virus-induced PABPC1 nuclear trafficking is RNase L-dependent. Collectively, our results suggest that SARS-CoV-2 Nsp15/EndoU activity contributes to the inhibition of OAS/RNase L activation.

###  rH234A infection does not induce SG formation in human lung cells

**Fig 7:**
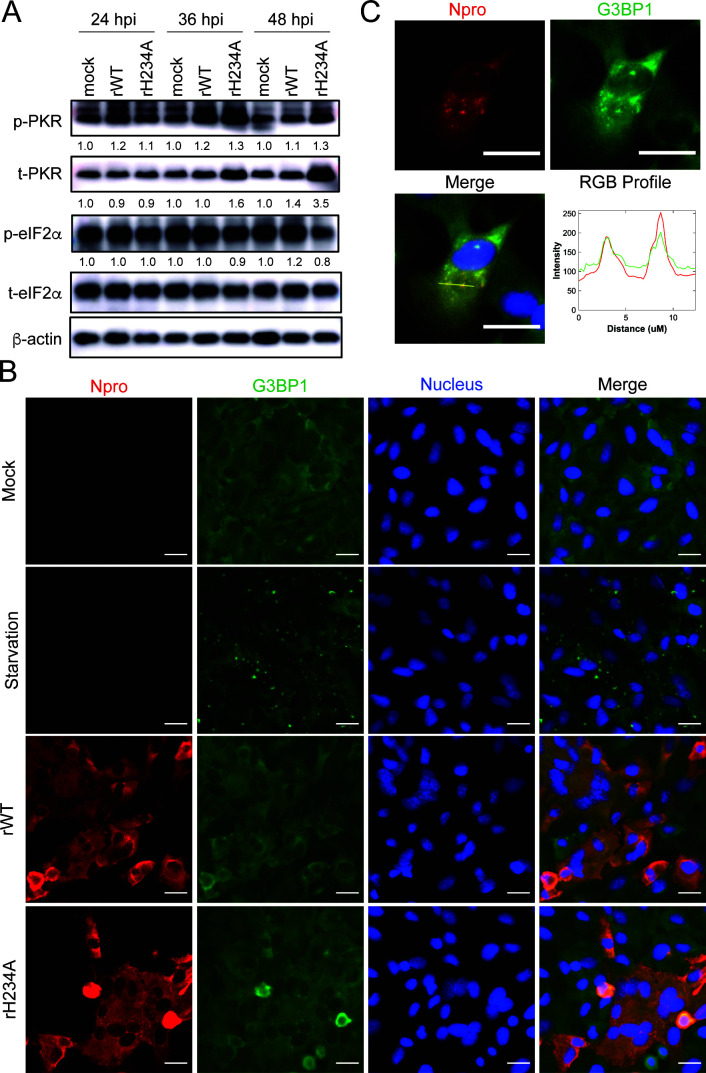
rH234A infection does not trigger stress granule formation in human lung cells. (**A**) Immunoblotting p-PKR, t-PKR, p-eIF2α, t-eIF2α, and β-actin of A549-A cells infected with 5 MOI of either rWT or rH234A and harvested at the indicated HPIs. The band intensities were measured with ImageJ. The relative protein levels were calculated as the band intensity relative to β-actin compared to the mock groups. (**B**) Immunofluorescence assay of viral N protein (N pro, red) and host G3BP1 protein (green). A549-A cells were cultured with 2% fetal calf serum media (Starvation) or infected with rWT or rH234A at 5 MOI and fixed at 24 HPI. The N protein and G3BP1 were detected with specific antibodies, and the nucleus was stained with Hoechst 33342. Scale bar: 20 µM. (**C**) Some cells in **B** show colocalization of the N protein and G3BP1. The RGB profile shows the fluorescent intensity signals of the N protein and G3BP1. Scale bar: 20 µM. Data in this figure are representative of at least two independent experiments.

Next, we investigated whether SARS-CoV-2 Nsp15/EndoU antagonizes activation of the PKR/eIF2α pathway, which can lead to global translational arrest and the formation of stress granules (SGs). We first tested whether the PKR/eIF2α pathway in A549-A cells is functional by examining the phosphorylation of PKR (p-PKR) and phosphorylation of eIF2α (p-eIF2α) upon transfection with poly(I:C). As shown in Fig. S7A, robust phosphorylation of PKR and eIF2α was detected in transfected cells compared to the untransfected control. We then examined the levels of p-PKR and p-eIF2α in A549-A cells infected with either virus with two different MOIs. At an MOI of 0.1, we did not observe an evident increase in p-PKR or p-eIF2α level in either virus-infected cells compared to the mock-infected controls (Fig. S7B and C). At an MOI of 5, increased levels of total PKR (t-PKR) and p-PKR levels were detected in rH234A-infected cells at 36 and 48 HPI compared to the rWT-infected cells (Fig. 7A). However, the p-eIF2α and total eIF2a (t-eIF2α) levels were not noticeably changed in either virus-infected cells compared to the mock-infected cells. We postulate that since PKR is an ISG, the increased PKR levels in rH234A-infected cells are possibly due to the elevated IFN response triggered by rH234A infection (Fig. 4A and B), thereby leading to increased p-PKR levels. However, the increased p-PKR levels did not result in greater eIF2α phosphorylation. These results suggest that despite the presence of marked amounts of intracellular viral dsRNA, neither rWT nor rH234A infection is sufficient to induce eIF2α phosphorylation.

Since phosphorylated eIF2α is required to halt protein synthesis and form SGs—cytoplasmic, membraneless compartments that assemble in response to stress stimuli like starvation or viral infection—we next assessed whether SGs were induced during infection. As shown in Fig. 7B, compared to mock cells cultured in a media containing 10% fetal calf serum (FCS), cytoplasmic G3BP1-positive puncta (a marker for SGs, green) were readily detected in A549-A cells cultured in an FCS-reduced (2%) media, indicating SG formation stimulated by nutrient deprivation or starvation. Interestingly, G3BP1-positive puncta were not readily detected in either rWT- or rH234A-infected cells (N positive, red), indicating that neither rWT nor rH234A infection triggers evident SG formation. This result concurs with the absence of p-eIF2α elevation in infected cells described above, suggesting that additional mechanism(s) may play roles in suppressing eIF2α phosphorylation or SG formation. Recent studies have reported that SARS-CoV-2 N protein interacts with G3BP1 and blocks SG formation (46–48). Our IFA result indeed shows that in some cells, the N protein colocalizes with G3BP1 (Fig. 7C) and forms puncta with a smaller size than SGs (Fig. S7D), consistent with recent studies (46–48). Taken together, our results demonstrate that although p-PKR levels are elevated in rH234A-infected cells, the PKR/eIF2α pathway is only weakly activated, resulting in limited SG formation.

### The Nsp15/EndoU activity promotes efficient viral infection in the k18-hACE2 mouse

**Fig 8:**
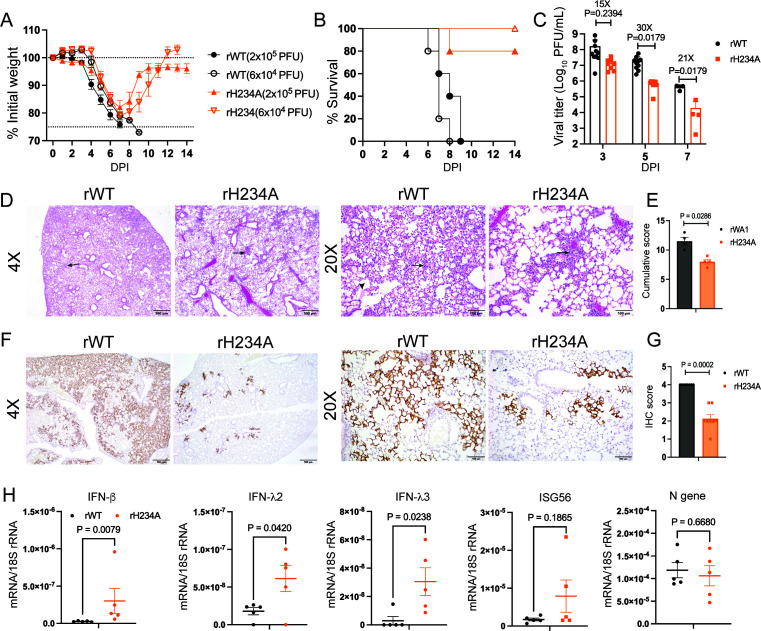
rH234A is attenuated in K18-ACE2 C57BL/6 mice. (**A and B**) Eight- to 10-week-old K18-ACE2 female mice were intranasally inoculated with a low dose (6 × 10^4^ PFU) or a high dose (2 × 10^5^ PFU) of rWT or rH234A. (**A**) Body weight change and (**B**) mortality rate were recorded daily for 14 days. Data in (**A**) are shown as mean ± SEM (*n* = 5 per group). The *P*-value of **B** was determined using a log-rank (Mantel-Cox) test. (**C**) Viral titers in lungs collected at 3, 5, and 7 dpi (*n* = 10 for 3 and 5 dpi, *n* = 4 for 7 dpi). The data were shown as mean ± SEM and analyzed with a two-way analysis of variance Tukey’s multiple comparison test. (**D**) Lungs collected at 7 dpi from mice infected with 2 × 10^5^ PFU were stained with hematoxylin and eosin. Photomicrographs of lungs demonstrate bronchointerstitial pneumonia (black arrow) and edema and fibrin (black arrowhead). Representative images are shown. (**E**) Lung sections (*n* = 4 per group) were scored for bronchointerstitial pneumonia, inflammation, perivascular infiltrates, and edema/fibrin deposition (each on a 0−4 scale). Bar graphs represent the cumulative lung pathology score. (**F**) Lung sections (*n* = 8 per group) collected at 5 dpi from mice infected with 2 × 10^5^ PFU were immunostained for SARS-CoV-2 nucleocapsid (brown color staining). Representative images are shown. (**G**) The immunostaining was scored based on the staining distribution for each mouse (*n* = 8 per group) on a scale of 0−4. Bar graphs represent the average scores for each group. Scale bars in **D and F**, 500 µm (4×) and 100 µm (20×). Data in **E and G** are mean ± SEM and analyzed with an unpaired nonparametric Mann-Whitney U-test (compare ranks). (**H**) RT-qPCR quantification of mouse *IFN-*β, *IFN-*λ*2/3*, *ISG56* mRNA, and viral N gene in mouse lungs collected at 1 dpi. Individual results and mean ± SEM (*n* = 5 mice per group) are shown and analyzed with unpaired *t*-test.

We next determined the role of Nsp15 EndoU activity *in vivo* using the k18-hACE2 C57BL/6 mouse model. To determine mouse morbidity and mortality, two independent infection experiments with two different virus dosages (2 × 10^5^ and 6 × 10^4^ PFU per mouse) were performed. In both experiments, intranasal inoculation with either virus led to similar body weight loss in mice starting at 4−5 days post-infection (dpi) (Fig. 8A). All of the rWT-infected mice succumbed to infection by 9 dpi; however, most of the rH234A-infected mice experienced a maximal 20% wt loss at 7 dpi and then started to gain body weight, resulting in 80%−100% survival rate (*P* < 0.0001) (Fig. 8B). We titrated the infectious viral titers of the lungs collected at 3, 5, and 7 dpi and found 15 ~ 30-fold lower lung titers in the rH234A-infected mice compared to the rWT-infected mice (Fig. 8C). These results indicate rH234A is attenuated in the k18-hACE2 mice. Further histopathological analysis revealed significantly reduced lung pathology in rH234A-infected mice at 7 dpi. Both groups exhibited interstitial pneumonia (black arrows), while fibrin deposition (black arrowheads) was observed in rWT-infected lungs at 20× magnification (Fig. 8D). However, the rWT virus induced a moderate accumulation of inflammatory exudates within the alveoli, whereas the rH234A virus resulted in largely open and clear alveolar spaces, with an overall lower cumulative pathological score (Fig. 8E). Immunohistochemistry (IHC) analysis of rWT-infected mouse lungs collected at 5 dpi showed strong staining for viral nucleocapsid protein (Fig. 8F). In contrast, lungs from rH234A-infected mice exhibited significantly reduced viral antigen staining, with a lower overall distribution of viral antigens (Fig. 8G). RT-qPCR analyses of mouse lungs collected at 1 dpi revealed that rH234A infection induced statistically higher mRNA levels of type I and III IFNs than rWT infection, suggesting that Nsp15/EndoU suppresses IFN response during infection in the lung (Fig. 8H). Collectively, these data demonstrate that rH234A is attenuated in the K18-hACE2 mice and suggest that the EndoU activity contributes to SARS-CoV-2 lung infection in this model.

## DISCUSSION

Current literature has documented that the conserved Nsp15/EndoU activity of CoVs is not directly involved in viral RNA synthesis but rather mediates the evasion of host antiviral responses, thereby promoting viral infection. Studies with MHV expressing enzymatically inactive Nsp15 mutants replicate similarly as the parental virus in immune-deficient cell lines but exhibited evident replication defects in immune-competent macrophages (11, 12). Similar findings were reported for other CoVs, such as PEDV, HCoV-229E, PDCoV, MERS-CoV, and IBV (12, 23–26). In this study, we generated recombinant EndoU-deficient SARS-CoV-2 (rH234A) and assessed the role of EndoU in the context of live viral infection. We found that the EndoU activity of SARS-CoV-2 is dispensable for viral propagation in Vero cells but crucial for infection in human lung cells (Fig. 1 to 3), consistent with the concept that the EndoU antagonizes the antiviral responses rather than directly participates in viral RNA synthesis. Two preprint studies by Chiu et al. (49) and Zhou et al. (50) reported similar replication defects of SARS-CoV-2 Nsp15 mutant viruses in human lung-derived cells. Interestingly, Otter et al. reported that a recombinant Nsp15 mutant virus (H234A) had comparable growth kinetics as the parental wild-type virus in human lung-derived A549-A and Calu-3 cell lines (51). We speculated that the MOI or the cell lines being used might contribute to this discrepancy. We tested multiple infection doses across several human lung-derived cell lines and found that the replication defect of rH234A was more pronounced at lower MOIs. Specifically, we observed 10- to 100-fold reductions in viral titers at low MOIs, compared to only 3 ~ 10-fold differences at high MOIs (Fig. 2). We extended our investigation to differentiated HBEC-ALI cultures and iPSC-derived AT2 organoids and found that rH234A exhibited a significant replication defect in both models. These findings further support the critical role of EndoU activity in promoting efficient SARS-CoV-2 replication in human lung epithelial cells.

Previous reports have documented that SARS-CoV-2 infection triggers delayed IFN response, which plays a critical role in the development of lung immunopathology in COVID-19 (52–54). Multiple SARS-CoV-2 proteins, including nonstructural and accessory proteins, have been implicated in contributing to the delayed IFN response (55). However, many of these investigations are based on overexpression systems, and only a few of them have been examined in the context of viral infection using recombinant viruses (56–61). Here, we demonstrated that the Nsp15 mutant viruses induced elevated mRNA levels of IFNs and ISGs in human lung cells and in mouse lungs, indicating that SARS-CoV-2 Nsp15/EndoU is a genuine IFN antagonist and suppresses IFN activation. This suppression not only promotes viral replication since inhibition of the IFN signaling using a JAK1/2 inhibitor could restore the replication defect of the Nsp15 mutant (51), but may also contribute to the delayed IFN response observed in COVID-19 patients (52–54).

In addition to preventing type I and III IFN responses, CoV Nsp15/EndoU has been implicated in preventing activation of the OAS/RNase L pathway. Infection of bone marrow-derived macrophages with EndoU-deficient murine hepatitis virus activates the OAS/RNase L pathway, resulting in massive rRNA degradation (11, 12). In the case of SARS-CoV-2, Otter et al. reported that infection of lung epithelial-derived cells with an EndoU-deficient virus did not lead to a noticeable increase in rRNA degradation compared to the WT control (51). The authors hypothesized that robust activation of the RNase L pathway by WT SARS-CoV-2 masked any further activation by the Nsp15 mutant. In our study, despite severely impaired replication, the rH234A mutant induced rRNA degradation at a rate comparable to that of the rWT virus during early infection. This suggests that rH234A was more effective at activating the RNase L pathway. Further immunofluorescence analysis revealed that rH234A infection resulted in approximately threefold more cells exhibiting PABPC1 nuclear accumulation—a hallmark of RNase L activation—supporting the idea that Nsp15/EndoU antagonizes this pathway. One notable observation from this IFA is that only a small fraction of infected cells displayed PABPC1 nuclear translocation (Fig. 6D; Fig. S4A), suggesting that the RNase L pathway remains inactive in the majority of infected cells, despite the presence of abundant dsRNA. This implies that, in addition to Nsp15, other viral factors may contribute to the suppression of this pathway. Indeed, the SARS-CoV-2 N protein has been reported to interact with dsRNA and inhibit the OAS/RNase L pathway (62, 63). In addition, the SARS-CoV-2 spike protein was recently shown to regulate dsRNA accumulation by modulating the expression of the p150 isoform of adenosine deaminase acting on RNA 1 (ADAR1) (64). ADAR1 is a dsRNA-binding protein and RNA-editing enzyme that catalyzes the deamination of adenosine to inosine (A-to-I editing) in RNA substrates (65, 66). Its p150 isoform was shown to modify cytoplasmic dsRNA and prevent activation of MDA5 and PKR (67). Whether and how these SARS-CoV-2 proteins, such as Nsp15/EndoU, N, and spike, act in concert to inhibit the OAS/RNase L pathway remains to be determined.

Another dsRNA-induced pathway being reportedly antagonized by CoV Nsp15/EndoU is the PKR/eIF2α pathway. We and others have reported that infection of EndoU-deficient MHV or IBV activated the PKR/eIF2α pathway, resulting in inhibition of protein translation and PKR-mediated apoptosis (11, 12, 24, 68). For MERS-CoV, inactivation of EndoU alone only induced mild phosphorylation of PKR during late infection, and no phosphorylation of eIF2α was detected (23). However, strong PKR activation was observed for a double-mutant virus expressing an inactive Nsp15 and with an NS4a deletion. For SARS-CoV-2, Otter et al. observed a modest increase in p-PKR and p-eIF2α levels in the Nsp15 mutant-infected A549-A cells, although the levels of total PKR and eIF2α were also increased (51). In our study, we also observed a weak increase of total PKR and p-PKR, but not total eIF2α and p-eIF2α, in the Nsp15 mutant-infected A549-A cells during late stage of infection with a high MOI condition (Fig. 7A; Fig. S7B and C). We postulate that the increased total PKR is possibly due to the activated type I/III IFN signaling, which can induce PKR expression, thereby resulting in more phosphorylated PKR through interferon-mediated mechanisms (69). Then we further determined whether the Nsp15 mutant infection activated the PKR/eIF2α pathway, in which case robust SG formation would be detected in infected cells. Several studies have reported that SARS-CoV-2 infection triggers very weak or inhibits SG formation (70–72), and Nsp15 was implicated in this inhibition as overexpressed Nsp15 inhibited SG formation induced by external stimuli (25, 68). Our results show, however, that Nsp15 mutant (rH234A) infection does not trigger evident SG formation in A549-A cells (Fig. 7B). Since the PKR/eIF2α pathway and SG formation are interconnected, we conclude that inactivation of Nsp15/EndoU activity is not sufficient to activate the PKR/eIF2α pathway and promote SG formation. Similar to MERS-CoV, we reason that additional viral factors may participate in the inhibition of this pathway. In fact, several studies have shown that the N protein inhibits PKR activation and its downstream events by sequestering dsRNA, preventing PKR autophosphorylation, and binding to p-PKR (63, 70–72). The N protein is also implicated in inhibiting the integrated stress response and SG formation by interacting with G3BP1 (47, 48, 71–74). Our IFA also revealed that in some SARS-CoV-2-infected cells, N protein colocalized with G3BP1 and formed cytoplasmic puncta with smaller sizes than SGs (Fig. 7C). Collectively, it is conceivable that SARS-CoV-2 employs multiple strategies, from limiting dsRNA accumulation or accessibility to inhibiting PKR activation or SG formation, to evade the antiviral effects of the PKR/eIF2α signaling.

Besides human lung-derived cell lines, we also employed other *in vitro* and *in vivo* models to investigate the role of Nsp15/EndoU in SARS-CoV-2 infection. Both HBEC-ALI cultures and AT2 organoids, which closely mimic the structure, differentiated cell types, and function of the human airway epithelium targeted by SARS-CoV-2, have been widely used in SARS-CoV-2 research to reveal host responses and viral infection dynamics (75–77). In this study, we found that Nsp15/EndoU was important for SARS-CoV-2 replication in these cultures derived from the lower respiratory tract (Fig. 3). Similarly, Otter et al. reported that the Nsp15 EndoU activity was important for SARS-CoV-2 infection in nasal epithelium ALI cultures (51). These studies together highlight the role of Nsp15/EndoU in SARS-CoV-2 infection in physiologically relevant *in vitro* models. More importantly, we further demonstrate a critical role for Nsp15 in SARS-CoV-2 infection *in vivo* using K18-hACE2 mice (Fig. 8), a widely used mouse model for SARS-CoV-2 pathogenesis studies (78, 79). While our manuscript was under revision, Caobi and colleagues reported that Nsp15 mutant viruses were less virulent in the K18-hACE2 mice (80), consistent with our findings. Notably, our additional data show that rH234A infection led to reduced lung pathology, decreased viral antigen staining, and an enhanced early IFN response in the lungs, further supporting our conclusion. Similar findings have also been reported in hamster models, where Nsp15 mutant viruses exhibited reduced viral load in respiratory tissues (49, 50).

In the real world, Nsp15/EndoU is also implicated in viral fitness. A computational analysis of 6.4 million SARS-CoV-2 genomes predicted that a T112I mutation of Nsp15 was independently associated with increased fitness (81). The T112I mutation is a marker of the Omicron lineage with 97% frequency (except BA.1). Biochemical characterization revealed that the T112I mutation increased the EndoU activity by 2.5-fold (31). Contrariwise, an H234Y mutation, first detected in a subclade of Delta and presented in 0.18% of SARS-CoV-2 genomes as of June 2024, resulted in an inactive form of EndoU like the H234A mutant (82). The results of these studies imply the role of Nsp15 EndoU activity in promoting the fitness of SARS-CoV-2 in humans. Taken together, the existing literature and our study demonstrate a critical role of Nsp15 in SARS-CoV-2 infection, underscoring its potential as a target for antiviral development.

## MATERIALS AND METHODS

### Ethical statement

All research personnel received rigorous biosafety, biosecurity, and biosafety level 3 (BSL3) training before participating in experiments. Personal protective equipment, including scrubs, disposable overalls, shoe covers, double-layered gloves, and powered air-purifying respirators, was used. Biosecurity measures are built in the environment through building and security systems and are reinforced through required training programs, standing meetings, and emergency exercises. The researchers involved in working with live viruses received the SARS-CoV-2 vaccines before the study was started. Finally, all researchers were medically cleared by the Oklahoma State University Occupational Health Program.

### Cell cultures

A Vero-E6 cell line, a gift from Dr. Susan Baker (Loyola University Chicago), was grown in Dulbecco’s modified Eagle medium (DMEM) (Corning, 10013CM) containing 10% heat-inactivated fetal bovine serum (FBS) (Gibco, 10-438-026), 1% penicillin/streptomycin (Pen/Strep) (Corning, 30002CI), 1% nonessential amino acid (NEAA) (Cytiva HyClone, SH30238.01). A Vero-E6 cell line expressing hACE2 and hTMPRSS2 (Vero-AT, NR-54970) and an A549 cell line expressing hACE2 (A549-A, NR-53821) were obtained from NIH-BEI Resources and cultured in DMEM, supplemented with 10% heat-inactivated FBS, 1× Pen/Strep, 1× NEAA, and 10 µg/mL puromycin (InvivoGen, ant-pr-1) to maintain the expression of hTMPRSS2 and hACE2. A Caco2 cell line expressing hACE2 and hTMPRSS2 (Caco2-AT) and an A549 cell line expressing hACE2 and hTMPRSS2 (A549-AT), gifts from Dr. Mohsan Saeed (Boston University), were cultured in DMEM with 10% FBS, 1× Pen/Strep, 1 µg/mL puromycin, and 1 µg/mL blasticidin (InvivoGen, ant-bl-05). A Calu-3 cell line was procured from ATCC (HTB-55) and cultured in DMEM containing 10% FBS and 1× Pen/Strep. A 293T cell line (ATCC, CRL-3216) was cultured in DMEM containing 10% FBS and 1× Pen/Strep. These cells were negative for mycoplasma contamination through periodically PCR detection.

A vial containing ≥ 5 × 10^5^ normal HBECs (Lonza, CC-2540) was thawed, and the cells were cultured in two T75 flasks using complete PneumaCult-Ex Plus Medium (STEMCELL Technologies, 05040) according to the manufacturer’s protocol. Once cells reached ~60% confluence, they were dissociated using TrypLE (Gibco, 12605028) and plated onto 0.4 μm pore Transwell inserts (Corning, 3470) at a density of 3.3 × 10^4^ cells per well. After reaching confluence, the cells were induced to differentiate at an ALI by removing the apical medium and replacing the basal compartment medium with the PneumaCult-ALI Medium (STEMCELL Technologies, 05001). The cells were differentiated for 4 weeks, with medium changes every other day to support the development of a fully differentiated epithelial layer.

The AT2 organoids (AO growth media) were derived from the human iPSC line BU3 NGST (a gift from Boston University Stem Cell Core Facility) through a directed differentiation approach, as we described previously (42). After 12–14 days of culture, the organoids were passaged, enzymatically digested using dispase II (Gibco, Catalog #17105), trypsinized, and reseeded in Matrigel (Corning, #354277). The cultures were maintained in alveolar organoid (AO) growth media with CEPT (50 nM chroman ^1^, 5 μM emricasan, 1× polyamines, and 0.^7^ μM trans-ISRIB) for the first 24 hours, followed by AO growth media without CEPT, refreshed every other day.

### Viruses

The recombinant SARS-CoV-2 viruses used in this study were generated using a reverse genetics system developed by the Baric group at the University of North Carolina at Chapel Hill and deposited to BEI Resources (NR-52281). The recombinant wild-type SARS-CoV-2 WA1 strain (rWT) was rescued from an infectious cDNA clone of the virus. To make mutant viruses, we used a Q5 site-directed mutagenesis kit (NEB, E0554S) to introduce alanine substitutions of His-234, His-14, and Trp-332 residues of Nsp15, and rescued viruses were designated rH234A, rH14A, and rW332A, respectively. These viruses were propagated and titrated in Vero-AT cells. Whole-genome sequencing was performed to confirm viral genotypes using the ARTIC method (83).

### Viral infection of cell cultures

All viral infections, plaque assays, and related manipulations were conducted in a BSL3 laboratory, adhering to established safety protocols and utilizing appropriate personal protective equipment. For cell line infections, viral inoculum at appropriate MOI was prepared in DMEM. Cells were washed once with cold phosphate-buffered saline (PBS, Corning, 46013CM) and incubated with the viral inoculum at 37°C for 1 hour, with gentle rocking every 15 min to facilitate adsorption. After incubation, the inoculum was removed and replaced with DMEM maintenance medium supplemented with 2% FBS. Culture supernatants were collected at designated time points for infectious virus quantification, and infected cells were harvested for total RNA or protein extraction.

For HBEC infection, differentiated cells in Transwell inserts were washed three times with 200 µL of Dulbecco’s PBS (DPBS; Gibco, 14190136), with each wash carried out at 37°C for 10 min. Viral inocula were prepared in DPBS (200 µL per insert) and inoculated onto the apical surface at 37°C for 3 hours. After incubation, the inocula were removed, and cells were washed once with 200 µL of DPBS. The medium in the basal compartment was replaced every other day during the infection course. To titrate the extracellular virus titer, 200 µL of DPBS was added to the insert, incubated at 37°C for 10 min, and collected for plaque assay. For RNA extraction, cells were lysed in RLT buffer (RNeasy Mini Kit, QIAGEN, 74106).

For AT2 organoid (AO) infection, 100 μL of Matrigel droplets containing approximately 1.5 to 2 million cells of AOs were used for infection. The AOs were separated from the Matrigel by incubating them with a cell recovery solution on ice for 45 min. Harvested AOs were rinsed with DMEM/F12 (Life Technologies Corporation, 11330-057) and used as apical-in AOs for viral infection. The apical-in AOs were then infected with recombinant SARS-CoV-2 at an MOI of 0.1 for 2 hours at 37°C. After 2 hours of absorption, the AOs were washed twice with DPBS. The apical-in AOs were reseeded in Matrigel droplets in a 12-well plate and cultured in AO growth medium at 37°C for 16, 24, 48, and 72 hours. Following incubation, the virus supernatants were collected, and the Matrigel was dissolved using a cell recovery solution to release the virus particles. Virus titers were measured from both the culture supernatant and Matrigel. The AO pellet was then treated with 1 mL of TRI-reagent (Molecular Research Center, TR118) to isolate RNA.

### Plaque assay

The collected samples of cell culture supernatants or tissue homogenates were titrated on Vero-AT cells. Samples were serially diluted in DMEM and inoculated onto Vero-AT cells seeded in 6- or 12-well plates. The cells were washed once with cold PBS and incubated with the diluted samples at 37°C for 1 hour, with gentle rocking every 15 min. After incubation, the inoculum was removed and replaced with a 0.6% agarose overlay (Fisherbrand, BP160-500) prepared in 1× DMEM supplemented with 2% FBS and 1× Pen/Strep. After 2 days of incubation, 4% formaldehyde was added directly onto the overlay and incubated at room temperature for 30 min to fix the cells. The overlay and fixative were then removed, and plaques were visualized by staining with 0.1% crystal violet solution (Fisher Chemical, C581-25). Each sample was assayed in technical duplicates, and viral titers were calculated as PFU per milliliter.

### Western blot

A total of 3 × 10^5^ cells were lysed in 100 μL of ice-cold lysis buffer (20 mM Tris-HCl, pH 7.5, 150 mM NaCl, 2 mM EDTA, 1% Triton X-100, 1× Halt protease inhibitor cocktail [Thermo Scientific, 87786], and 1× Halt phosphatase inhibitor cocktail [Thermo Scientific, 78420]) on ice for 10 min with occasional tilting. Lysates were mixed with 4× Laemmli buffer (62.5 mM Tris-HCl, pH 6.8, 8% SDS, 40% glycerol, 0.4% bromophenol blue, and 10% β-mercaptoethanol) at a 1:3 (vol/vol) ratio, heated at 100°C for 10 min, cooled on ice for 2 min, and centrifuged at 14,000 × *g* at 4°C for 5 min. Supernatants were subjected to SDS-PAGE using gels of appropriate acrylamide concentrations. Proteins were transferred to methanol-activated polyvinylidene difluoride (PVDF) membranes (Fisher, ISEQ00010) at 80V for 90 min. Then membranes were blocked in 5% non-fat milk or 5% bovine serum albumin (BSA; for phospho-protein immunoblotting) in 1× TBST (19.8 mM Tris-HCl, pH 7.6, 150 mM NaCl, 0.1% Tween 20) for 1 hour at room temperature and incubated with primary antibodies (Table 1) overnight at 4°C. After washing with 1× TBST (5 min × 4 times), membranes were incubated with appropriate secondary antibodies (Table 1) for 1 hour at room temperature. Following four washes with 1× TBST, detection was performed using ECL substrates (Fisher, PI34578) and chemiluminescence imaging. ImageJ was used for signal quantification. Membranes were stripped in stripping buffer (62.5 mM Tris-HCl, pH 6.8, 2% SDS, and 10% β-mercaptoethanol) at 55°C for 30 min, thoroughly washed with TBST, and re-blocked prior to re-probing.

**TABLE 1:** Sources and dilutions of antibodies used for Western blotting

Antibody	Source and catalog no.	Dilution
β-Actin	Genscript, A00702-100	1:3,000
RNase L	Cell signaling, 27281S	1:1,000
Phospho-eIF2α (Ser51)	Cell signal, 9721S	1:1,000
eIF2α	Cell signal, 9722S	1:1,000
Phospho-PKR (Thr446)	Fisher, PIMA532086	1:1,000
PKR	Cell signaling, 12297S	1:1,000
Goat anti-rabbit IgG	Fisher, OB4030-05	1:7,000
Goat anti-mouse IgG	Fisher, OB1010-05	1:7,000

### RNA extraction and real-time PCR quantification

Total RNA was extracted from cells lysed with the RLT buffer of the Qiagen RNeasy kit (Qiagen 74106) following the manufacturers’ protocols. A total of 500–1,000 ng of RNA (adjusted based on RNA concentration) was used for cDNA synthesis using the RT² HT First Strand Kit (QIAGEN 330411). Real-time quantitative PCR was performed using specific primers (Table 2) and PowerUp SYBR Green Master mix (Fisher, A25918) on QuanStudio 6 Pro (Thermo Fisher, A43160). Cycle threshold values were normalized to human glyceraldehyde-3-phosphate dehydrogenase (GAPDH) or mouse 18S rRNA using the 2^-ΔCt^ method.

**TABLE 2:** qPCR primers

Species	Gene	Strand	Nucleotide sequences (5´−3´)
Human	GAPDH	Forward	GTCTCCTCTGACTTCAACAGCG
		Reverse	ACCACCCTGTTGCTGTAGCCAA
	IFN-β1	Forward	CTTGGATTCCTACAAAGAAGCAGC
		Reverse	TCCTCCTTCTGGAACTGCTGCA
	IFN-λ1	Forward	AACTGGGAAGGGCTGCCACATT
		Reverse	GGAAGACAGGAGAGCTGCAACT
	IFN-λ3	Forward	TCGCTTCTGCTGAAGGACTGCA
		Reverse	CCTCCAGAACCTTCAGCGTCAG
	ISG 56	Forward	GCCTTGCTGAAGTGTGGAGGAA
		Reverse	ATCCAGGCGATAGGCAGAGATC
Mouse	18S rRNA	Forward	TCGGAACTGAGGCCATGATT
		Reverse	TTTCGCTCTGGTCCGTCTTG
	IFN-β1	Forward	GCCTTTGCCATCCAAGAGATGC
		Reverse	ACACTGTCTGCTGGTGGAGTTC
	IFN-λ2	Forward	CCAGTGGAAGCAAAGGATTGCC
		Reverse	TCAGGTCCTTCTCAAGCAGCCT
	IFN-λ3	Forward	CCAGTGGAAGCAAAGGATTGCC
		Reverse	GCACCTCATGTCCTTCTCAAGC
	ISG56	Forward	TACAGGCTGGAGTGTGCTGAGA
		Reverse	CTCCACTTTCAGAGCCTTCGCA
SARS-CoV-2	N gene	Forward	AAGCTGGACTTCCCTATGGTG
		Reverse	CGATTGCAGCATTGTTAGCAGG

### Ribosomal RNA degradation analysis

Intracellular RNA was harvested with RLT buffer and extracted using the Qiagen RNeasy kit (QIAGEN). For RNA integrity assessment, 400 ng of total RNA in 6 μL H_2_O was analyzed via TapeStation analysis (Agilent Technologies) at the One Health Innovation Laboratory, Oklahoma State University.

### Generation of RNase L knockout cells using CRISPR/Cas9

Two published guide RNA (gRNA) sequences for generating an RNase L knockout cell line are 5´-caccgTTTGAGGCGAAAGACAAAGG-3´ and 5´-aaacCCTTTGTCTTTCGCCTCAAAc-3´ (84). Specific overhang sequences (F: caccg...; R: aaac...c) were added to the gRNA oligos. Forward (F) and reverse (R) oligos (1 μL of 10 μM each) were annealed in a 50 μL reaction, incubated at 37°C for 30 min, heated to 95°C for 1 min, and gradually cooled to 25°C (decreasing 1.6°C per cycle per min). The annealed oligos were ligated into lentiCRISPRv2 puro (Addgene, #98290) using T4 ligase (NEB M0202L) and BsmBI-V2 (NEB R0739L) in a 10 μL reaction, with the following ligation cycle: 37°C for 10 min (10 cycles), 16°C for 5 min (10 cycles), 55°C for 20 min, 80°C for 20 min, and then held at 4°C. The ligation mixture was transformed into competent *Escherichia coli* (Top10 cells) by heat shock, plated on Luria-Bertani (LB) agar containing ampicillin, and incubated overnight. Colonies were picked and cultured, and plasmid DNA was extracted using miniprep (Promega A1222). Diagnostic digestion with KpnI-HF (NEB R3142S) and AgeI (NEB R3552S) confirmed the correct constructs (expected size 621 bp), which were then sent for sequencing for further verification. For lentiviral packaging, 293T cells were seeded at 6 × 10^5^ cells per well in a six-well plate and transfected with 2 μg each of lentiCRISPRv2, psPAX2 (Addgene, #12260), and pHEF-VSV-G (Addgene, #22501) using Lipofectamine 3000 (Fisher, L3000008). Viral supernatants were harvested at 48 and 72 hours post-transfection, clarified by centrifugation, and stored at −80°C. Then A549-A cells (60%–70% confluence) were infected with virus (1 mL) in the complete DMEM with 7 µg/mL polybrene (Fisher, TR1003G). After 48 hours of incubation at 37°C, stable clones were selected by treatment with 6 μg/mL puromycin in complete DMEM, which was increased to 10 μg/mL on subsequent days. Knockout efficiency was confirmed by Western blot (WB) analysis, and validated clones were maintained in 10 μg/mL puromycin for further experiments.

### Immunofluorescence assay

A549-ACE2 cells were seeded at a density of 5 × 10^4^ cells per well on sterile coverslips placed in a 24-well plate. The following day, cells were infected with icWA1 or icNsp15 mutant virus at an MOI of 5. At 24 hours post-infection, cells were washed twice with cold PBS and fixed with cold paraformaldehyde fixative solution (Thermo Fisher Scientific, AAJ61984AP) for 20 min at room temperature. After fixation, cells were briefly washed three times with cold PBS. Fixed cells were then permeabilized and blocked for 30 min at room temperature using PBS-TBF (1× PBS + 0.1% Triton X-100 + 1% BSA + 10% FBS). Cells were then incubated overnight at 4°C with the primary antibody diluted in 1% BSA in PBST (Table 3). The next day, cells were washed three times with cold PBS (5 min per wash), followed by incubation with the secondary antibody in 1% BSA for 1 hour at room temperature in the dark. Subsequently, cells were washed three times with PBS (5 min per wash). To prepare slides for imaging, one drop of mounting medium (Thermo Fisher Scientific, P36983) was applied directly onto a microscope slide, and a coverslip was carefully placed onto the mountant. Images were captured using a fluorescence microscope (CELENA S Digital Imaging System).

**TABLE 3:** Sources and dilutions of antibodies used for immunofluorescence assay

Antibody	Source and catalog no.	Dilution
SARS-CoV-2 nucleocapsid antibody [HL448]	Genetex, GTX635686	1:100
dsRNA, mouse monoclonal K1	Exalpha, 10020200	1:250
G3BP, mouse monoclonal [2F3]	Abcam, ab56574	1:500
PABP, rabbit polyclonal	Abcam, ab21060	1:500
Goat anti-rabbit IgG (H + L), Alexa Fluor 647	Fisher, A21244	1:1,000
Goat anti-mouse IgG (H + L), Alexa Fluor 488	Fisher, A11029	1:1,000

### Mouse experiments

Eight- to 9-week-old female K18-hACE2 C57BL/6 mice (strain #: 034860) were procured from The Jackson Laboratory. The mice were briefly anesthetized with isoflurane and then inoculated intranasally with DMEM as a mock, or rWA1 or rH234A, in a total volume of 50 μL DMEM. Mouse body weight and health were monitored daily. Per Institutional Animal Care and Use Committee (IACUC)-approved protocol, mice that experienced a 25% body weight loss were humanely euthanized as a predefined endpoint. Weight loss and mortality data were analyzed using Prism 10. Three control mice were euthanized at 3 dpi, while groups of 10 mice from each infection group were euthanized at 3 and 5 dpi, and 4 mice at 7 dpi for necropsy. The left lungs were collected in pre-filled bead tubes (Fisher Scientific, 15-340-153) containing 1 mL of DMEM for viral load determination and RT-qPCR analysis. For histopathological analysis, the remaining lung tissues were fixed in 25 mL of zinc-buffered formalin solution for a minimum of 2 days, following an approved Institutional Biosafety Committee (IBC) protocol, before being removed from animal biosafety level 3 containment.

### Lung histology and IHC

Fixed tissues were trimmed and processed using a Sakura Tissue-Tek VIP 6 AI tissue processor (Sakura Finetek USA, Inc., CA) on a delayed short cycle program and embedded in paraffin (Leica Surgipath Paraplast Infiltration and Embedding Medium; Leica Biosystems). Paraffin blocks were cut into 4 μm-thick sections and mounted on VistaVision HistoBond adhesive glass slides from VWR (Radnor, PA). Hematoxylin and eosin staining was performed following standard operating procedures with the Sakura Finetek DRS601 (Sakura Finetek USA, Inc., CA). For IHC, slides were rehydrated with water, following HIER (heat-induced epitope retrieval) performed at 95°C for 20 min in Citrate Unmasking Solution (H-3300, Vector Laboratories, Newark, CA). SARS-CoV-2 nucleocapsid antibody (HL448) (Genetex, GTX635686) was diluted 1:5,000 in TBS-Tween 20 buffer with 10% normal goat serum, and slides were incubated for 1 hour at room temperature. Slides were washed in TBS-Tween 20 buffer and then quenched of endogenous peroxidase using 0.3% H_2_O_2_ for 10 min. Slides were washed, and detection was carried out using VECTASTAIN Elite ABC-HRP Kit, Peroxidase (Rabbit IgG) (Vector Laboratories, PK-6101) per the manufacturer’s instructions. Hematoxylin diluted 1:10 was used as a counterstain. Stained tissue sections were evaluated by a board-certified veterinary pathologist for three parameters: presence of edema or hyaline membranes, perivascular lymphoid inflammation, and interstitial inflammation. Edema or hyaline membranes were evaluated using a distribution-based ordinal scoring on a scale of 0 to 5 with 0, none; 1, <5%; 2, 6%−25%; 3, 26%−50%; 4, 51%−75%; and 5, >75% of tissue affected. Perivascular lymphoid inflammation and interstitial pneumonia were evaluated using a severity-based ordinal scoring system on a scale of 0 to 4: 0 (absent), 1 (minimal), 2 (mild), 3 (moderate), and 4 (severe). An accumulative score is calculated by summing the ordinal scores of the three parameters. IHC was scored using a distribution-based ordinal scoring of 0 to 5 with 0, none; 1, <5%; 2, 6%−25%; 3, 26%−50%; 4, 51%−75%; and 5, >75% of tissue affected.

### Statistical analysis

The data generated in this study are analyzed using GraphPad Prism version 10.0.0 (GraphPad Software, Boston, MA, USA). The statistical tests for specific data sets are described in the corresponding figure legend. Statistical comparisons were performed on ΔCt values to satisfy assumptions of normality and equal variance. For clarity, relative expression (2^–ΔCt^) is presented in figures. All experiments were repeated independently at least twice. Results are presented as means ± standard errors of the means or standard deviation. *P*-values of ≤0.05 were considered statistically significant (*, *P *≤ 0.05; **, *P *≤ 0.01; ***, *P* ≤ 0.001; ****, *P* ≤ 0.0001; ns, not significant).

## Data Availability

All associated data are available upon request. The viral strains used in this study are available through a material transfer agreement.

## SUPPLEMENTAL MATERIAL

Supplemental figures jvi.01175-25-s0001.docx Figures S1 to S7.

ASM does not own the copyrights to Supplemental Material that may be linked to, or accessed through, an article. The authors have granted ASM a non-exclusive, world-wide license to publish the Supplemental Material files. Please contact the corresponding author directly for reuse.
