# Supplementary material for: SARS-CoV-2 Nsp15 endoribonuclease subverts host defenses to enhance viral fitness in lung cells
Source: J Virol. 2025 Aug 21;99(9):e01175-25. doi: 10.1128/jvi.01175-25 (PMC12456009; doi:10.1128/jvi.01175-25)
Supplement: Supplemental figures — Figures S1 to S7. [file jvi.01175-25-s0001.docx]

**Supplementary Figures**

| 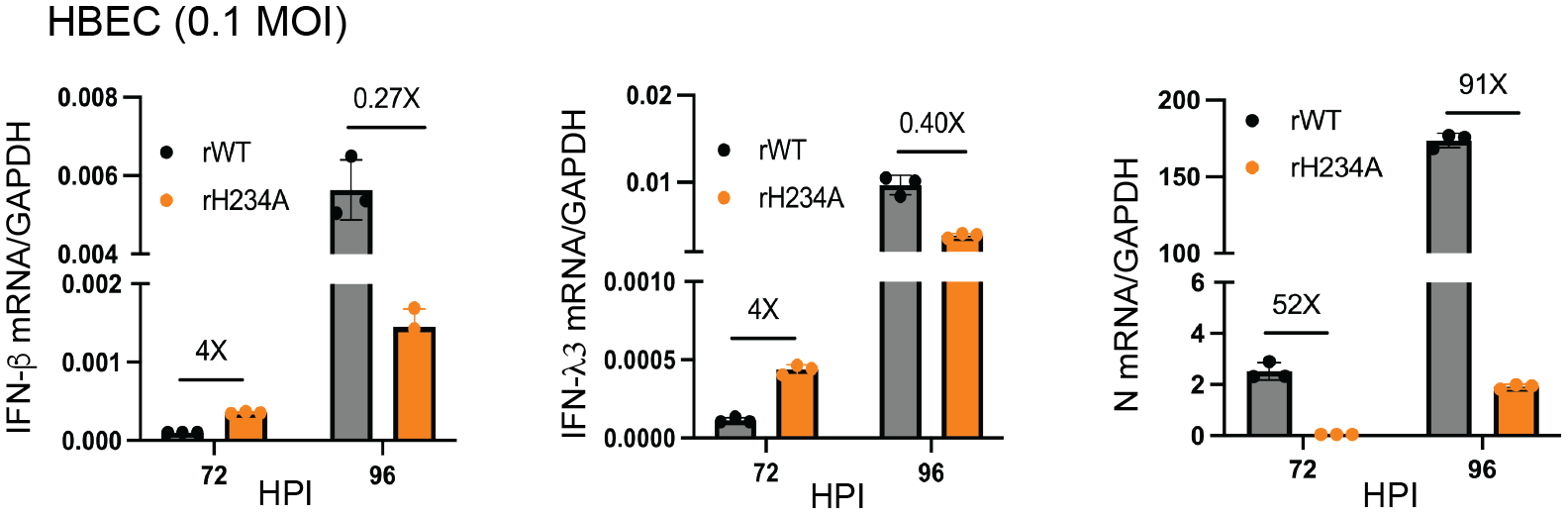 |
| --- |
| **Fig. S1. Impaired rH234A replication elicits comparable type I/III IFN responses as rWT infection in HBEC-ALI cultures**. Quantification of *IFN-β, IFN-λ3,* and viral N gene levels in HBEC-ALI cultures infected with 0.1 MOI of rWT or rH234A by RT-qPCR. Data are shown as mean ± SD (n = 3) and were analyzed using a two-way ANOVA with Tukey’s multiple comparisons test. *, P < 0.05; **, P < 0.01; ***, P < 0.001; ****, P < 0.0001. |

| 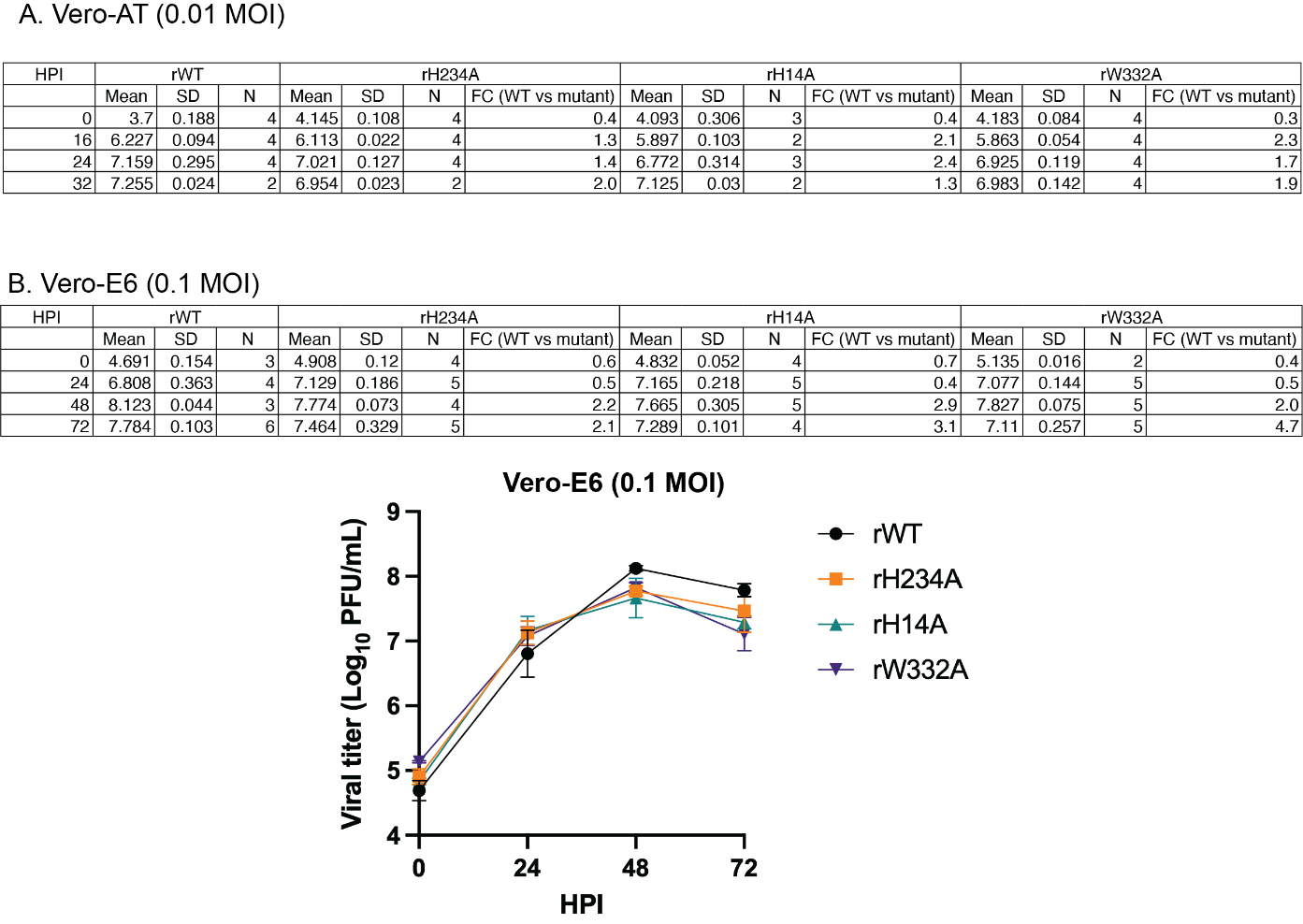 |
| --- |
| **Fig. S2. Growth kinetics of SARS-CoV-2 Nsp15 mutants in Vero cells.** (A) Growth kinetics of rH234A, rH14A, rW332A mutants in Vero-AT cells. (B) Growth kinetics of rH234A, rH14A, rW332A mutants in Vero-E6 cells. Data are representative of at least two independent experiments and are shown as mean ± SD (n = 4-6). |

| 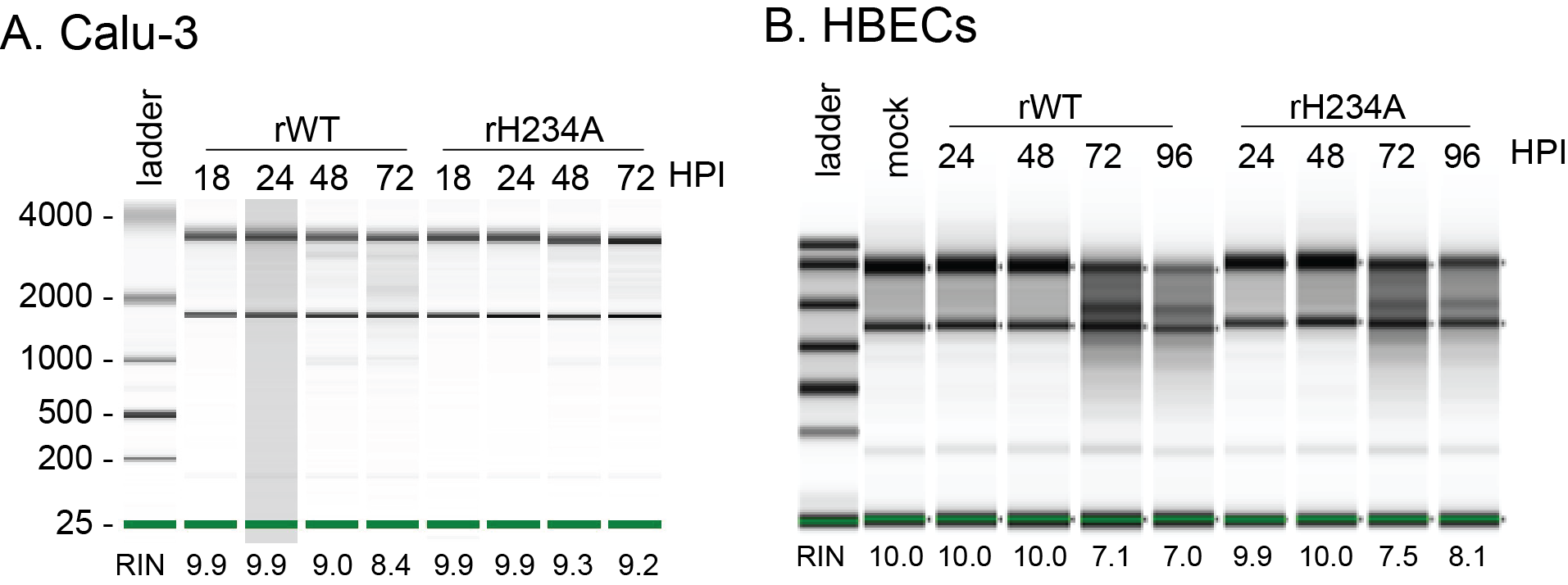 |
| --- |
| **Fig. S3. rH234A infection activates the OAS/RNase L pathway at levels comparable to rWT infection in Calu-3 cells and HBECs.** (A) RNA integrity analysis of Calu-3 cells infected with rWT or rH234A at 0.1 MOI. (B) RNA integrity analysis of HBEC cells infected with rWT or rH234A at 0.1 MOI. The cells were harvested at the indicated times for total RNA extraction and subsequent TapeStation analysis. RNA Integrity Number (RIN): a higher value indicates better RNA integrity. |

| 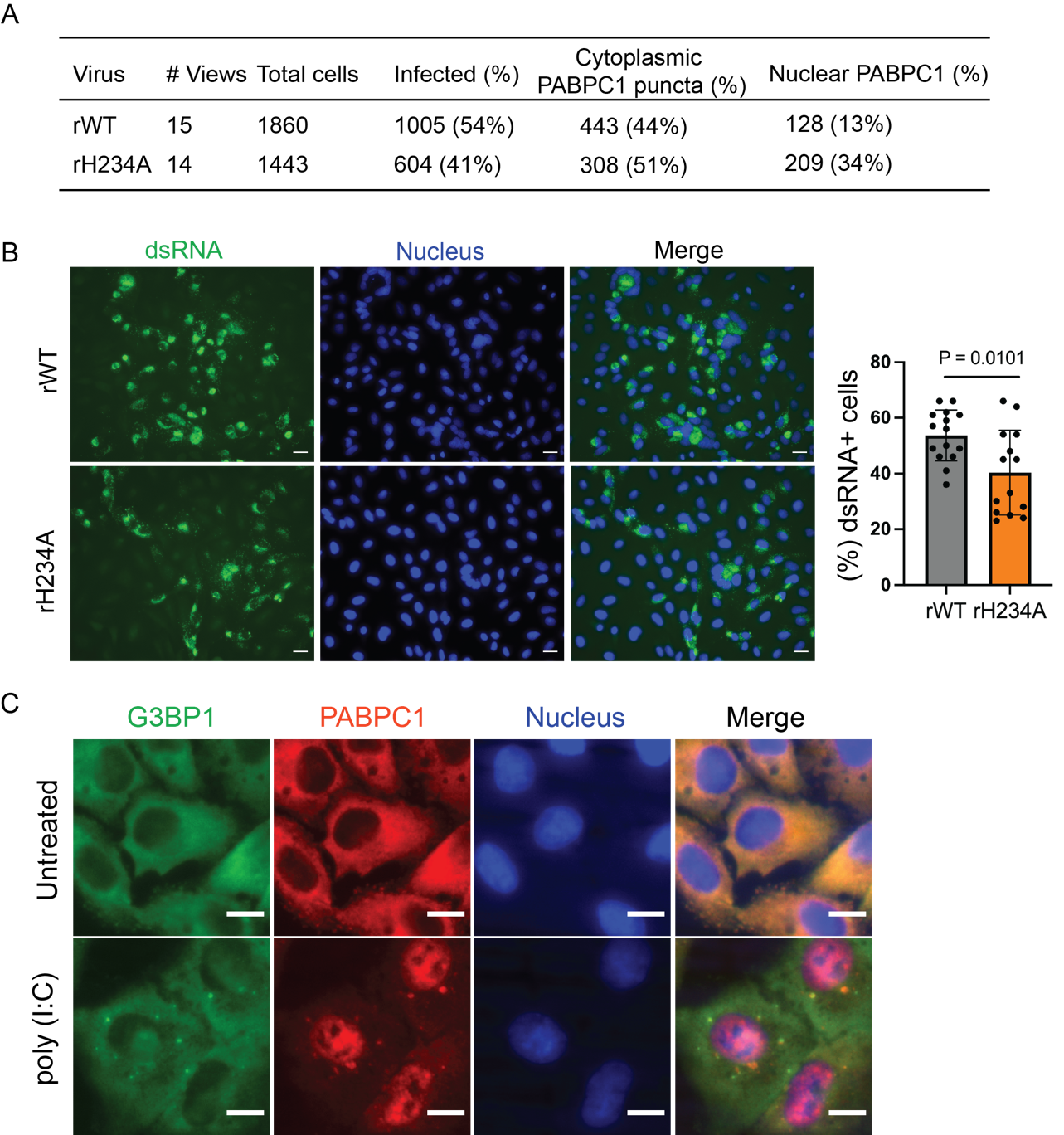 |
| --- |
| **Fig. S4. PABPC1 subcellular localization in rWT- and rH234A-infected A549-A cells.** (A) Percentage of infected cells and nuclear/cytoplasmic PABPC1 localization ratios for rWT and rH234A. (B) (Left) Representative immunofluorescence images of A549-A cells infected with rWT or rH234A (5 MOI, 24 hpi), stained with dsRNA antibodies (green) and nuclei (blue). (Right) Quantification of infected cells (dsRNA-positive cells/total cells). Data are shown as mean ± SEM (n = 14-15) and analyzed using Unpaired t test with Welch’s correction. Scale bar: 20 µm. (C) Immunofluorescence analysis of G3BP1 (green) and PABPC1 (red) localization in A549-A cells transfected with 500 ng/mL poly(I:C) for 6 hours prior to fixation. Stress granules were labeled with host G3BP1 (green). Scale bar: 20 µm. |

| 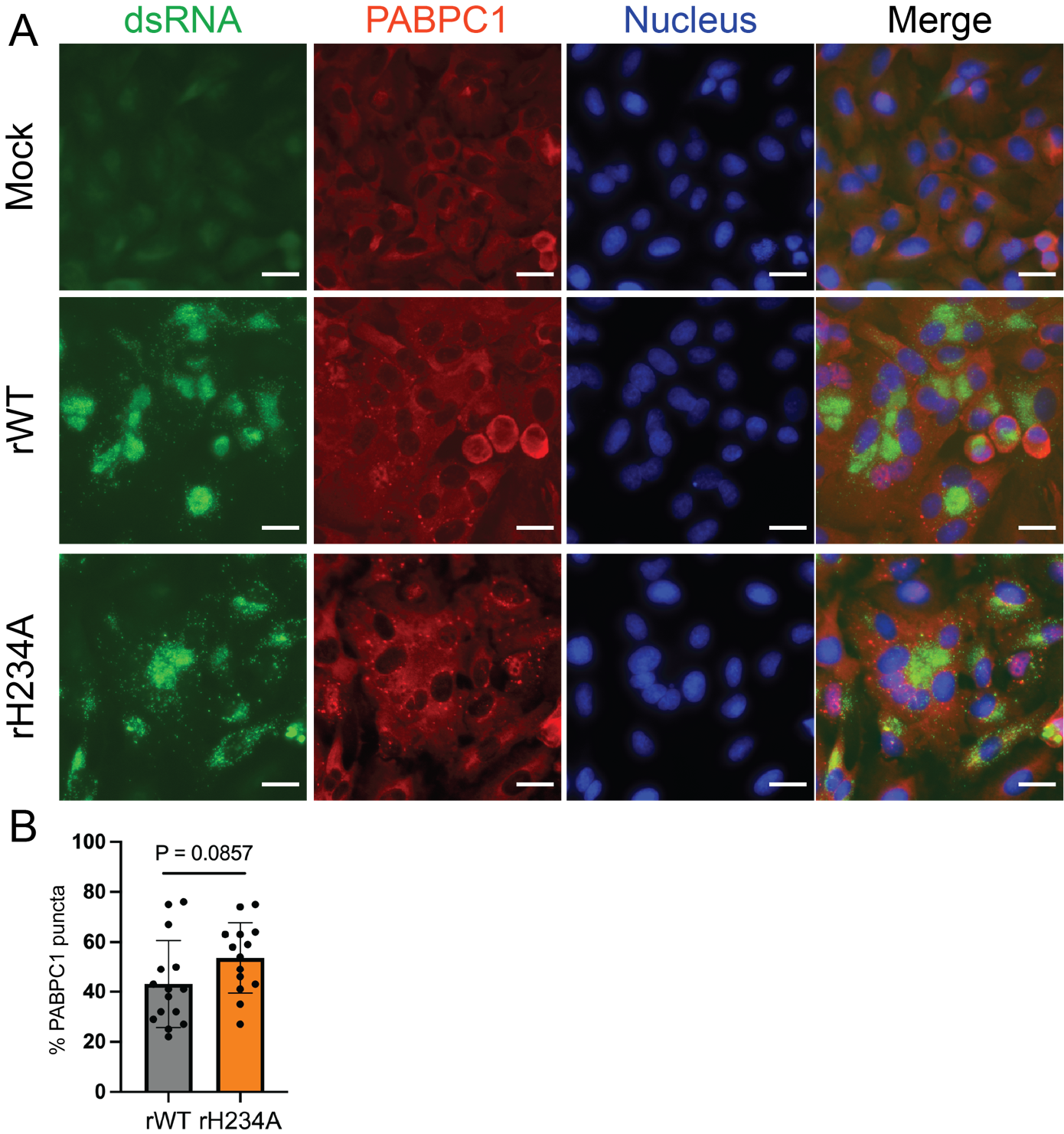 |
| --- |
| **Fig. S5. PABPC1 formed cytoplasmic punta in rWT- and rH234A-infected A549-A cells.** (A) Representative images of infected A549-A cells (MOI 5, 24 HPI) stained with PABPC1 (red), dsRNA (green), and nuclei (blue). (B) Quantification of cytoplasmic PABPC1 puncta (rWT vs. rH234A). Data are shown as mean ± SEM (n = 14-15) and analyzed using Unpaired t test with Welch’s correction. Scale bar: 20 µm. |

| 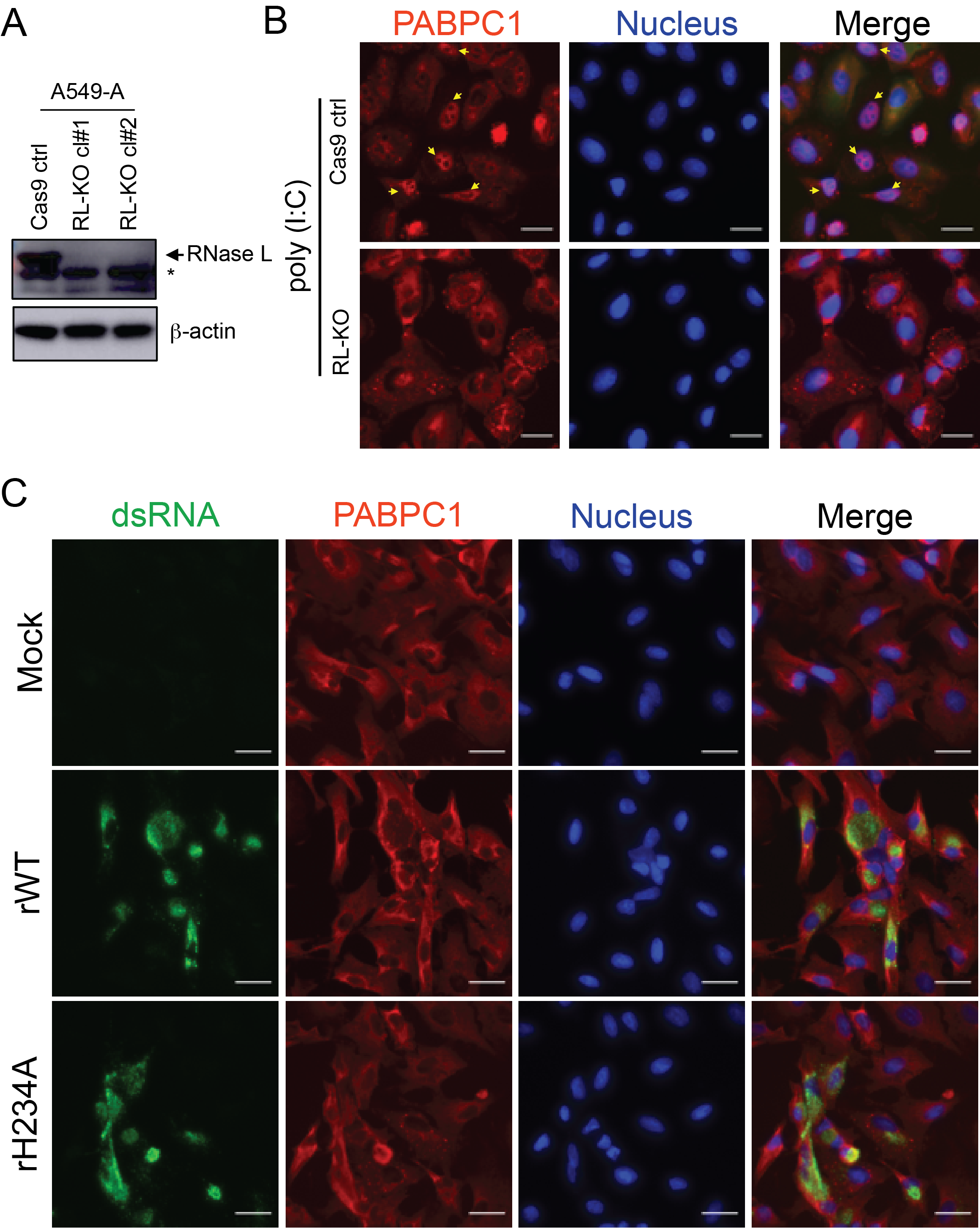 |
| --- |
| **Fig. S6. RNase L is required for SARS-CoV-2-induced PABPC1 nuclear trafficking.** (A) Western blot verification of RNase L expression in CRISPR-Cas9 knockout A549-A cells. (B) Representative images of PABPC1 staining in the Cas9 control and RNase L knockout cells (RL-KO) after poly(I:C) transfection (500 ng/mL). (C) Representative images of PABPC1 and dsRNA staining in the RNase L knockout cells after rWT or rH234A infection (5 MOI, 24 HPI). |

| 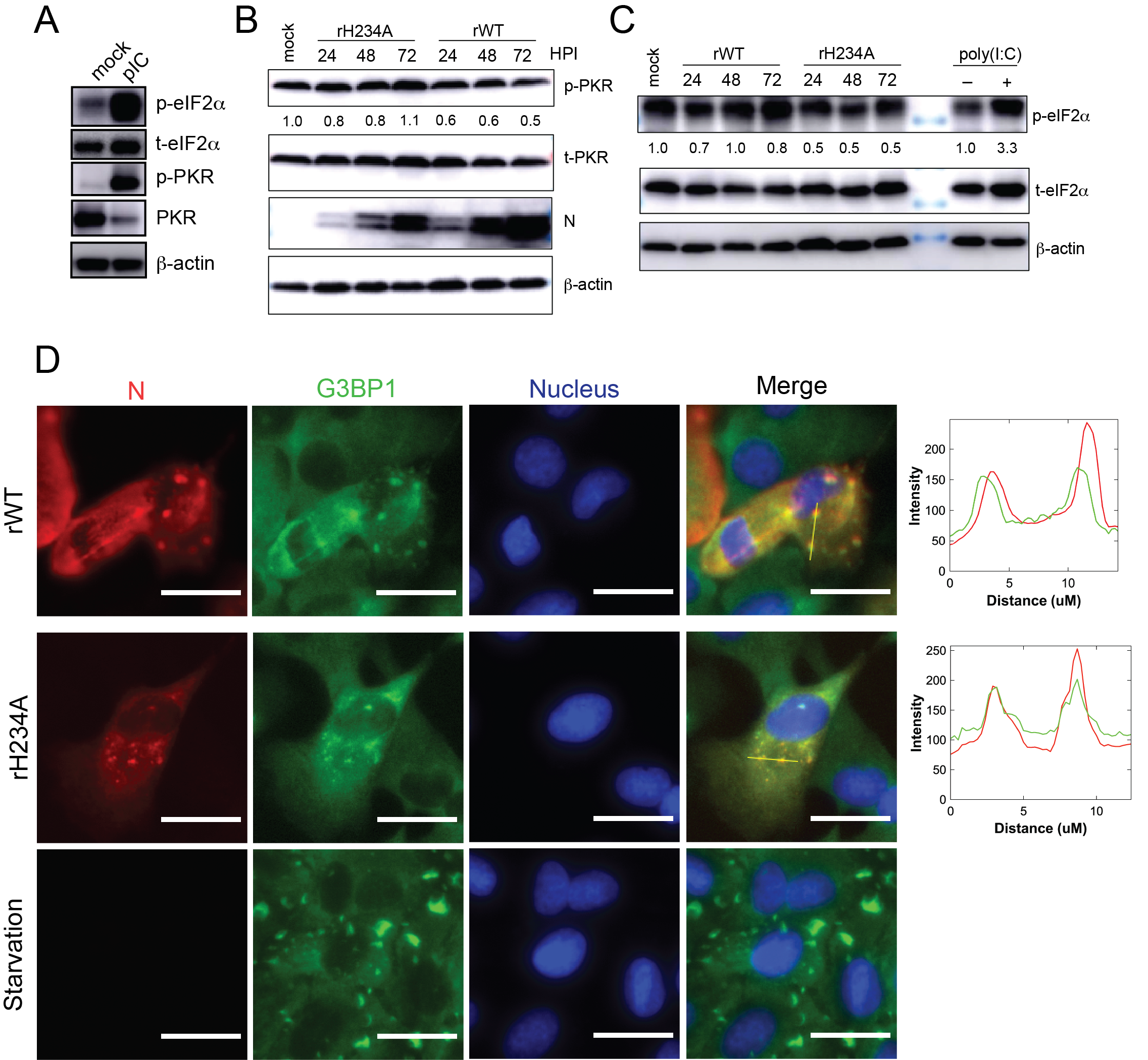 |
| --- |
| **Fig. S7. Assessment of PKR/eIF2α activation and SG formation in SARS-CoV-2 infection.** (A) Western blotting of phosphorylated PKR (p-PKR), total PKR (t-PKR), phosphorylated eIF2α (p-eIF2α), total eIF2α (t-eIF2α), and β-actin of A549-A cells transfected with 500 ng/mL poly(I:C) and harvested at 6 hours post-transfection. (B) Western blotting of p-PKR, t-PKR, viral N protein (N), and β-actin of A549-A cells infected with 0.1 MOI of either rWT or rH234A and harvested at the indicated HPI. (C) Western blotting of p-eIF2α, t-eIF2α, and β-actin of A549-A cells infected with 0.1 MOI of either rWT or rH234A and harvested at the indicated HPI. (D) Representative IFA images of viral N protein and G3BP1 localization in infected A549-A cells. Viral N protein (N, red) and G3BP1 protein (green), RGB profiling graphs of N and G3BP1 are shown. |
